# Safety and efficacy of double-balloon catheter for cervical ripening: a Bayesian network meta-analysis of randomized controlled trials

**DOI:** 10.1186/s12884-022-04988-2

**Published:** 2022-09-06

**Authors:** Ge Zhao, Guang Song, Jing Liu

**Affiliations:** 1 Department of Obstetrics, The First Hospital of China Medical University, No. 155 Nanjing North Street, Heping District, Shenyang, Liaoning Province 110001 China; 2 Department of Ultrasound, Shengjing Hospital of China Medical University, Shenyang, China

**Keywords:** Cervical ripening, Labor induction, Double-balloon catheter, Foley, Dinoprostone, Misoprostol, Meta-analysis

## Abstract

**Background:**

Various methods are used for cervical ripening during the induction of labor. Mechanical and pharmacological methods are commonly used for cervical ripening. A double-balloon catheter was specifically developed to ripen the cervix and induce labor; however, the efficacy of the double-balloon catheter in cervical ripening compared to other methods is unknown.

**Methods:**

We searched five databases and performed a Bayesian network meta-analysis. Six interventions (double-balloon catheter, Foley catheter, oral misoprostol, vaginal misoprostol, dinoprostone, and double-balloon catheter combined with oral misoprostol) were included in the search. The primary outcomes were cesarean delivery rate and time from intervention-to-birth. The secondary outcomes were as follows: Bishop score increment; achieving a vaginal delivery within 24 h; uterine hyperstimulation with fetal heart rate changes; need for oxytocin augmentation; instrumental delivery; meconium staining; chorioamnionitis; postpartum hemorrhage; low Apgar score; neonatal intensive care unit admission; and arterial pH.

**Results:**

Forty-eight randomized controlled trials involving 11,482 pregnant women were identified. The cesarean delivery rates of the cervical ripening with a double-balloon catheter and oral misoprostol, oral misoprostol, and vaginal misoprostol were significantly lower than cervical ripening with a Foley catheter (OR = 0.48, 95% CI: 0.23–0.96; OR = 0.74, 95% CI: 0.58–0.93; and OR = 0.79, 95% CI: 0.64–0.97, respectively; all *P* < 0.05). The time from intervention-to-birth of vaginal misoprostol was significantly shorter than the other five cervical ripening methods. Vaginal misoprostol and oral misoprostol increased the risk of uterine hyperstimulation with fetal heart rate changes compared to a Foley catheter. A double-balloon catheter with or without oral misoprostol had similar outcomes, including uterine hyperstimulation with fetal heart rate changes compared to a Foley catheter.

**Conclusion:**

Double-balloon catheter did not show superiority when compared with other single method in primary and secondary outcomes of labor induction. The combination of double-balloon catheter with oral misoprostol was significantly reduced the rate of cesarean section compared to Foley catheter without increased risk of uterine hyperstimulation with fetal heart rate changes, which was shown in oral or vaginal misoprostol.

**Supplementary Information:**

The online version contains supplementary material available at 10.1186/s12884-022-04988-2.

## Introduction

Labor induction is a common obstetric procedure; 20 to 30% of deliveries are induced worldwide [1]. Successful induction of labor depends on the status of the cervix at the time of induction. A poor Bishop score has been shown to be associated with an unacceptably high induction failure rate [2]. Medical interventions are necessary to induce cervical ripening prior to initiation of labor if the Bishop score is ≤ 6 [3–5].

Methods of cervical ripening can be broadly categorized into mechanical and pharmacological methods [4, 6]. Mechanical methods apply pressure from inside the cervical canal to force dilation. The local pressure stimulates the release of prostaglandins (PGs), which facilitate cervical remodeling. Foley catheters and transcervical double-balloon catheters are the two major devices utilized for mechanical dilation [7]. Compared with the unilateral pressure of a single-balloon catheter, the double-balloon catheter offers an improved mechanism of dilation between the internal and external cervical os [8]. There are a variety of pharmaceutical agents available for cervical ripening, including PGs, oxytocin, estrogens, and mifepristone. PGE_2_ cervical ripening with controlled-release dinoprostone inserts has gained widespread use in clinical practice. Misoprostol, a synthetic structural analog of PGE_1_, has been shown to be effective in labor induction and is often used as an off-label drug for inducing labor.

To determine if the double-balloon catheter was better than other methods, recent clinical trials have been designed to compare the efficacy and safety with a Foley catheter [9], dinoprostone insert [10], and misoprostol [11]; however, the results have not led to a consensus. We therefore conducted a network meta-analysis (NMA) comparing the double-balloon catheter with four commonly used cervical ripening in labor induction methods among pregnant women in the third trimester with intact membranes. The purpose of this study was to provide a comprehensive overview of the available evidence involving the use of a double-balloon catheter for cervical ripening in clinical practice.

## Methods

The pre-registered protocol was implemented in the PROSPERO database (CRD42022317381). This NMA was reported in accordance with the PRISMA guidelines (Supplemental Table S1).

### Search strategy

The PubMed, MEDLINE, Embase, ClinicalTrials.gov, and Cochrane Library databases were searched on March 18, 2022 to identify the relevant studies by two investigators. The keywords in the search strategy were as follows: “cervical ripening” or “labor, induced”; and “double-balloon catheter” or “single-balloon catheter/Foley catheter” or “dinoprostone” or “misoprostol” (Supplemental Table S2). Additionally, we searched the references of articles to further identify literature that met the criteria.

### Data extraction and extraction

Original studies were eligible if the following criteria were met: (I) randomized controlled trial (RCT) studies; (II) full text available in English; and (III) the efficacy and safety of different interventions (double-balloon catheter, single balloon catheter/Foley catheter, oral misoprostol, vaginal misoprostol, 10-mg controlled-release dinoprostone vaginal insert, and double-balloon catheter combined with misoprostol/dinoprostone) for cervical ripening in women with an unfavorable cervix and with intact membranes were assessed.

Original studies were ineligible for the following reasons: (I) reviews, observational studies, case control studies, abstracts, letters, or case reports; (II) trials including women whose pregnancies were ≤ 28 weeks gestational age, non-cephalic presentations, multiple pregnancies, or a previous cesarean section(s); (III) other forms of dinoprostone (gel or tablet); or (IV) laboratory animal studies. In the case of several publications from the same study, the study with the greatest number of cases and most relevant information was included.

The first author, year of publication, treatment groups, and number of participants in each group, age (years), nulliparity, gestational age (weeks), balloon volume (mL), misoprostol route and dose, and outcomes were extracted from the eligible studies.

### Outcomes

The primary outcomes were cesarean delivery rate and the time from intervention-to-birth. The secondary outcomes included achieving vaginal delivery within 24 h, Bishop score increment, uterine hyperstimulation with fetal heart rate changes, oxytocin augmentation, instrumental delivery, meconium-stained amniotic fluid, maternal adverse events (chorioamnionitis and postpartum hemorrhage), and neonatal adverse events (low Apgar score, neonatal intensive care unit admission, and arterial pH).

### Statistical analysis

Prior to analysis, the risk of trial bias was assessed for the included studies using the Cochrane Collaboration’s tool. The mean difference (MD) and 95% confidence interval (CI) were the time from intervention-to-birth and Bishop score increment. Odds ratios (ORs) were used to report the cesarean delivery rate, achieving vaginal delivery within 24 h, uterine hyperstimulation with fetal heart rate changes, oxytocin augmentation, instrumental delivery, and meconium-stained amniotic fluid. We evaluated the efficacy and safety of different interventions for cervical ripening in women with an unfavorable cervix and intact membranes using an NMA. In this Bayesian NMA, random-effects and consistency models were used to analyze data and carry out the NMA (4 chains, 50,000 iterations, and 20,000 per chain). We assessed inconsistencies using the node-splitting method, and inconsistencies are reported by the Bayesian *P* values. An overall grading of the quality of evidence was conducted using the GRADE system. To rank the outcomes, we used the surface under the cumulative ranking curve (SUCRA) as an indicator (worst: 0; best:1) for each intervention. We analyzed the symmetry of a comparison-adjusted funnel plot to evaluate possible small sample effects and used Begg’s and Egger’s tests to evaluate publication bias in the included studies. A *p* value < 0.05 was considered statistically significant for asymmetry. All analyses were conducted using the “gemtc” package of R (version 4.0.2; R Foundation, Vienna, Austria) and Stata (version 16.0; StataCorp, College Station, TX, USA).

## Results

### Baseline characteristics of included studies

**Fig. 1:**
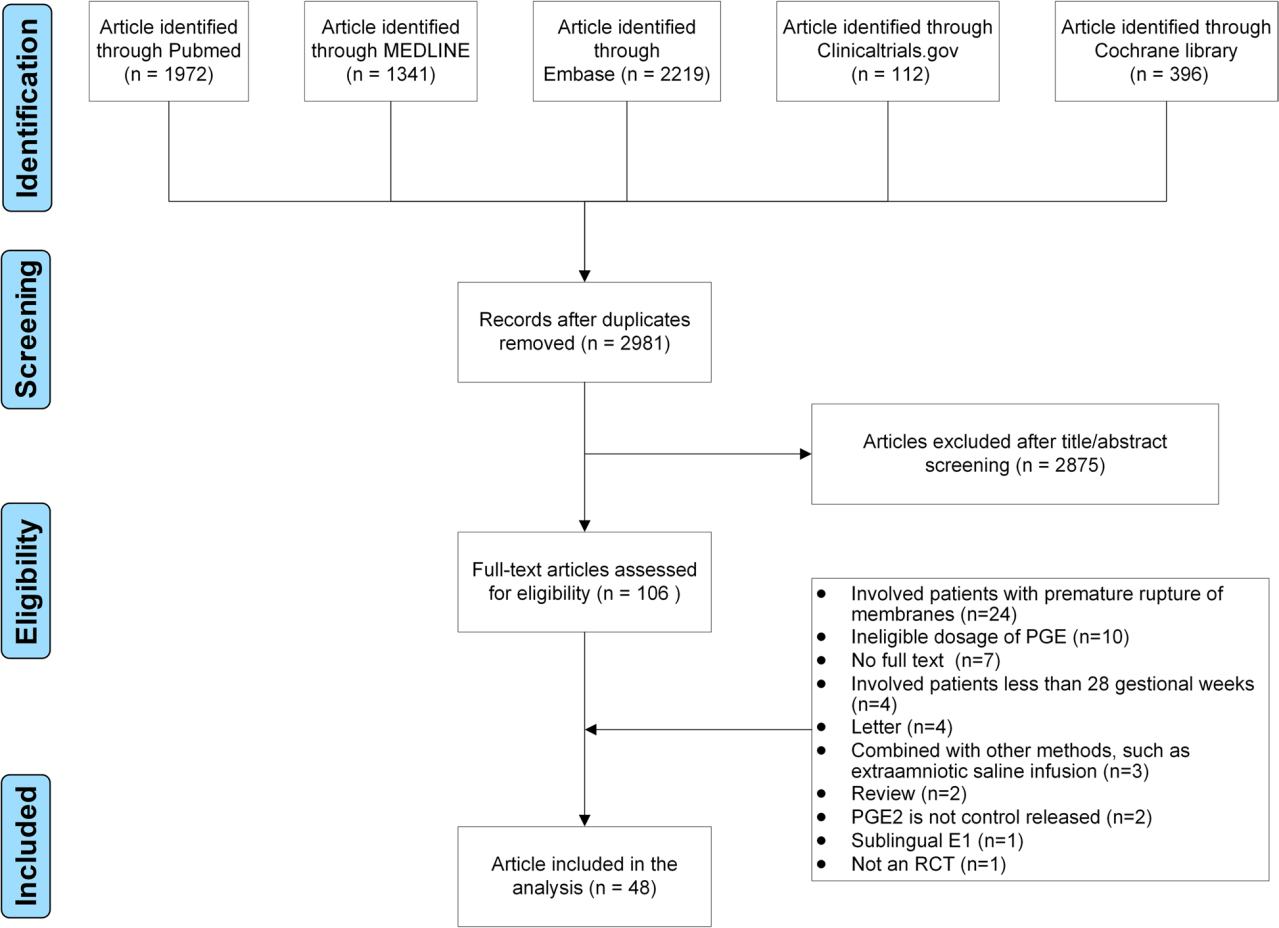
Flow chart of study selection

Our exhaustive search strategy retrieved 2,981 potentially relevant publications from six databases. After screening and reading the full-text articles, 48 RCTs were included in our final analyses (Fig. 1) [10–57]. These RCTs were conducted between 1997 and 2021 (Table 1) and were carried out in Asia (China, India, Iran, Israel, Saudi Arabia, Sri Lanka, Thailand, and Turkey), Australia, Europe (France, Germany, Italy, the Netherlands, and the UK), and North America (the USA and Canada). Six types of intervention were assessed, including oral misoprostol, vaginal misoprostol, dinoprostone, Foley catheter, double-balloon catheter, and double-balloon catheter with oral misoprostol. All of the studies were two-arm with 11,482 pregnant women. The balloon volume, misoprostol dose, and outcomes of each study are shown in Table 1. The evaluation of bias risk for all RCTs is presented in Supplemental Figure S1 and S2.

**Table 1:** Characteristics of included studies

Author, year	Country	Groups	Numbers	Age (years)	Nulliparity (%)	Gestational age (weeks)		Balloon volume (mL)	Misoprostol dose	Outcomes
**Wing, 1997 **[12]	USA	Dinoprostone	98	NR	42.9	39.2 ± 2.3		-	-	①②③⑤⑥⑧⑨⑫⑬
		Vaginal misoprostol	99	NR	48.5	39.5 ± 2.4			25 mcg every 4 h up to 6 doses	
**Bennett, 1998 **[13]	Canada	Vaginal misoprostol	102	28.7 ± 4.9	72.5	40.6 ± 1.2		-	50 mcg every 4 h up to 5 doses	①⑥⑦⑪⑫
		Oral misoprostol	104	27.5 ± 5.0	66.3	40.8 ± 1.1			50 mcg every 4 h up to 9 doses	
**Wing, 1999 **[14]	USA	Oral misoprostol	110	NR	48.2	39.2 ± 1.7		-	50 mcg every 4 h up to 6 doses	①②③⑤⑥⑧⑨⑪⑫⑬
		Vaginal misoprostol	110	NR	48.2	38.6 ± 2.0			25 mcg every 4 h up to 6 doses	
**Fisher, 2001 **[15]	Canada	Vaginal misoprostol	64	27.0 ± 4.5	56.2	41.0 ± 2.3		-	50 mcg every 3 h up to 48 h	①②⑤⑥⑦⑧⑬
		Oral misoprostol	62	27.0 ± 6.0	64.5	41.0 ± 1.5			50 mcg every 6 h up to 48 h	
**Khoury, 2001 **[16]	USA	Dinoprostone	39	28.1 ± 7.0	59.0	39.9 ± 1.4		-	-	①②③⑤⑥⑧⑪⑫⑬
		Vaginal misoprostol	79	29.7 ± 6.3	62.0	40.0 ± 1.2			35 mcg every 4.5 h up to 6 doses or 50 mcg every 4.5 h up to 6 doses	
**Kwon, 2001 **[17]	Canada	Oral misoprostol	78	27.2 ± 5.4	56.4	40.3 ± 1.8		-	50 mcg every 6 h up to 8 doses	①②⑥⑪
		Vaginal misoprostol	82	27.6 ± 5.1	52.4	40.3 ± 1.7			50 mcg every 6 h up to 8 doses	
**Sciscione, 2001 **[18]	USA	Foley catheter	58	25.1 ± 6.9	70.6	> 28		30 mL	-	①②③⑤⑧
		Vaginal misoprostol	53	25.9 ± 6.9	71.7	> 28		-	50 mcg every 4 h up to 6 doses	
**Shetty, 2001 **[19]	UK	Oral misoprostol	122	28.0 ± 6.8	59.8	41.0 ± 1.3		-	50 mcg every 4 h up to 5 doses	①③⑤⑥⑦⑧⑬
		Vaginal misoprostol	123	28.0 ± 7.8	61.8	41.0 ± 1.3			50 mcg every 4 h up to 5 doses	
**le Roux, 2002 **[20]	South Africa	Vaginal misoprostol	120	27.9 (mean)	43.3	39 (mean)		-	50 mcg every 6 h up to 4 doses	①③⑥⑬
		Oral misoprostol	120	28.1 (mean)	36.0	38.3 (mean)			50 mcg every 6 h up to 4 doses	
		Dinoprostone	240	27.6 (mean)	42.0	39 (mean)			-	
**Chung, 2003 **[21]	USA	Vaginal misoprostol	49	26.3 ± 6.8	67.3	39.8 ± 2.3		-	25 mcg every 3 h up to 6 doses	①⑤⑥⑦⑧⑨⑩⑫⑬
		Foley catheter	54	26.5 ± 6.0	61.1	40.0 ± 2.1		30 mL	-	
**Nopdonrattakoon, 2003 **[22]	Thailand	Oral misoprostol	53	24.9 ± 5.5	NR	39.0 ± 1.0		-	50 mcg every 4 h up to 6 doses	①③⑤⑥⑦⑧
		Vaginal misoprostol	53	25.3 ± 5.5	NR	39.1 ± 1.1			50 mcg every 4 h up to 6 doses	
**Ramsey, 2003 **[23]	USA	Dinoprostone	38	26.7 ± 3.6	NR	39.3 ± 1.3		-	-	①②④⑧⑩⑬
		Vaginal misoprostol	38	27.9 ± 4.6	NR	39.3 ± 1.6			50 mcg every 6 h up to 2doses	
**Shetty, 2003 **[24]	UK	Oral misoprostol	51	28.6 ± 6.2	56.9		40.7 ± 1.3	-	100 mcg every 4 h up to 5 doses	①②⑤⑥⑦⑧⑬
		Vaginal misoprostol	50	28.0 ± 5.5	56.0		40.9 ± 1.1		25 mcg every 4 h up to 5 doses	
**Paungmora, 2004 **[25]	Thailand	Oral misoprostol	75	29.1 ± 4.9	78.7		41.0 ± 1.3	-	100 mcg every 6 h up to 8 doses	①②⑥⑧⑩⑬
		Vaginal misoprostol	76	28.2 ± 4.7	73.7		40.5 ± 1.0		50 mcg every 6 h up to 8 doses	
**Rozenberg, 2004 **[26]	France	Vaginal misoprostol	70	29.0 ± 5.2	62.9		41.3 ± 1.6	-	50 mcg every 6 h up to 1 dose in the first day and 50 mcg every 4 h up to 3 doses in the second day	①②⑤⑥⑧⑨⑩⑪⑫⑬
		Dinoprostone	70	29.0 ± 3.7	67.1		41.4 ± 2.1			
**Adeniji, 2005 **[27]	Nigeria	Vaginal misoprostol	50	30.2 ± 3.5	52.0		39.9 ± 1.7	-	50 mcg every 6 h up to 4 doses	①⑦⑧⑫⑬
		Foley catheter	46	30.5 ± 3.8	43.5		40.2 ± 1.3	30 mL	-	
**Afolabi, 2005 **[28]	Nigeria	Vaginal misoprostol	29	NR	44.8		NR	-	100 mcg once	①②⑤⑥
		Foley catheter	28	NR	46.2		NR	30 mL	-	
**Gelisen, 2005 **[29]	Turkey	Vaginal misoprostol	100	25.9 ± 5.9	46.0		41.0 (mean)	-	50 mcg every 6 h up to 4 doses	①⑤⑧⑪⑫⑬
		Foley catheter	100	24.4 ± 4.1	47.0		41.0 (mean)	50 mL	-	
**Owolabi, 2005 **[30]	Nigeria	Vaginal misoprostol	60	29.6 ± 0.8	19.0		40.7 ± 0.2	-	50 mcg every 6 h up to 2 doses	①③⑤⑥⑦⑧⑩⑪⑫⑬
		Foley catheter	60	31.1 ± 0.8	22.8		40.3 ± 0.3	30 mL	-	
**Ayaz, 2009 **[31]	Saudi Arabia	Oral misoprostol	44	34.3 (mean)	NR		NR	-	50 mcg every 4 h up to 4 doses	③⑤⑧⑪⑫⑬
		Vaginal misoprostol	44	35.9 (mean)	NR		NR		50 mcg every 4 h up to 4 doses	
**Ozkan, 2009 **[32]	Turkey	Vaginal misoprostol	56	NR	51.8		> 37	-	50 mcg every 4 h up to 5 doses	①②③⑤⑥⑧⑪⑬
		Dinoprostone	56	NR	57.1		> 37		-	
**Pennell, 2009 **[33]	Australia	Double-balloon catheter	107	27.0 ± 6.0	100.0		40.0 ± 1.5	80 mL + 80 mL	-	①②③⑦⑩⑪
		Foley catheter	110	26.0 ± 7.0	100.0		40.0 ± 1.5	30 mL		
**Cromi, 2011 **[34]	Italy	Foley catheter	265	32.1 ± 4.7	69.1		39.8 ± 1.9	50 mL	-	①②③④⑤⑥⑦⑪⑬
		Dinoprostone	132	31.0 ± 4.9	67.4		39.8 ± 2.0	-		
**Roudsari, 2011 **[35]	Iran	Vaginal misoprostol	49	24.3 ± 4.0	NR		39.8 ± 1.4	-	25 mcg every 4 h up to 6 doses	①②⑧
		Foley catheter	59	24.2 ± 5.0	NR		40.0 ± 0.9	50 mL	-	
**Salim, 2011 **[36]	Israel	Foley catheter	145	28.8 ± 6.1	53.1		39.2 ± 1.4	60 mL	-	①②③④⑦
		Double-balloon catheter	148	29.2 ± 5.5	52.7		39.0 ± 1.6	80 mL + 80 mL		
**Cromi, 2012 **[37]	Italy	Double-balloon catheter	105	34.0 ± 5.8	78.1		40.4 ± 2.0	50 mL + 50 mL	-	①②③④⑥⑦⑩⑪⑬
		Dinoprostone	103	33.0 ± 6.3	72.8		40.6 ± 2.1	-		
**Kandil, 2012 **[38]	Egypt	Foley catheter	50	28.0 ± 3.8	100		41.3 ± 0.3	30 mL	-	①②⑤⑥⑦⑧⑨
		Vaginal misoprostol	50	28.9 ± 4.3	100		41.4 ± 0.3	-	25 mcg every 4 h up to 4 doses	
**Jozwiak, 2013 **[39]	Netherlands	Foley catheter	107	30.5 ± 4.0	72.0		39.1 ± 1.9	30 mL	-	①②⑤⑥⑦⑩⑪⑫⑬
		Dinoprostone	119	31.7 ± 5.2	70.0		39.8 ± 2.1	-		
**Ugwu, 2013 **[40]	Nigeria	Foley catheter	45	28.9 ± 4.3	44.0		40.7 ± 1.5	30 mL	-	①⑥⑦⑫⑬
		Vaginal misoprostol	45	27.1 ± 4.9	42.0		40.2 ± 1.7	-	25 mcg every 4 h up to 6 doses	
**Edwards, 2014 **[41]	USA	Foley catheter	185	28.0 ± 6.4	57.3		39.1 ± 1.4	30 mL	-	①②③⑥⑧⑨⑪⑬
		Dinoprostone	191	26.9 ± 5.9	66.5		39.2 ± 1.5	-		
**Jozwiak, 2014 **[42]	Netherlands	Foley catheter	56	31.0 ± 5.0	66.1		39.1 ± 2.2	30 mL	-	①②⑤⑥⑦⑩⑪⑬
		Vaginal misoprostol	64	32.3 ± 5.2	64.1		39.8 ± 2.1	-	25 mcg every 4 h up to 3 doses	
**Suffecool, 2014 **[43]	USA	Dinoprostone	31	28.0 ± 7.1	100		40.2 ± 1.5	-	-	①②③⑥⑦⑪
		Double-balloon catheter	31	27.5 ± 6.4	100		40.9 ± 1.1	80 mL + 80 mL		
**Wang, 2014 **[44]	China	Double-balloon catheter	67	27.9 ± 3.9	100		39.3 ± 2.1	80 mL + 80 mL	-	①③④⑥⑩⑪⑬
		Dinoprostone	59	27.8 ± 3.4	100		39.0 ± 1.3	-		
**Chavakula, 2015 **[45]	India	Vaginal misoprostol	46	25.1 ± 4.7	69.6		37.8 ± 1.2	-	25 mcg every 6 h up to 3 doses	①②③⑤⑥⑨⑪⑬
		Foley catheter	54	24.3 ± 3.9	63.0		37.7 ± 1.1	30 mL	-	
**Du, 2015 **[46]	China	Double-balloon catheter	76	28.5 ± 4.6	89.5		40.5 ± 0.9	80 mL + 80 mL	-	①③④⑤⑥⑦⑧⑩⑪⑫⑬
		Dinoprostone	79	27.3 ± 3.3	91.1		40.6 ± 0.8	-		
**Ezechukwu, 2015 **[47]	Nigeria	Oral misoprostol	70	27.2 ± 4.5	62.9		40.6 ± 1.5	-	50 mcg every 6 h up to 4 doses	①⑦⑩⑪⑫⑬
		Vaginal misoprostol	70	28.2 ± 3.7	60.0		40.7 ± 1.6		50 mcg every 6 h up to 4 doses	
**Kehl, 2015 **[11]	Germany	Double-balloon catheter with oral misoprostol	162	30.0 ± 6.0	53.7		40.4 ± 1.1	80 mL + 80 mL	50 mcg orally every 4 h up to 3 doses in the first 24 h then 100 mcg orally every 4 h up to 3 doses in the next 24 h and then 100 mcg vaginally every 4 h up to 3 doses	①②⑥⑦⑧⑨⑪
		Oral misoprostol	151	30.0 ± 6.5	60.9		40.3 ± 1.1	-	50 mcg orally every 4 h up to 3 doses in the first 24 h then 100 mcg orally every 4 h up to 3 doses in the next 24 h and then 100 mcg vaginally every 4 h up to 3 doses	
**Noor, 2015 **[48]	India	Vaginal misoprostol	60	25.1 ± 2.8	41.7		39.1 ± 1.4	-	25 mcg every 4 h up to 6 doses	①②⑤⑥⑬
		Foley catheter	44	25.6 ± 4.1	31.8		39.4 ± 1.2	50 mL	-	
**Shechter-Maor, 2015 **[49]	Israel	Dinoprostone	26	28.5 ± 5.3	50.0		40.0 ± 1.0	-	-	①②⑥⑦⑧⑬
		Double-balloon catheter	26	28.5 ± 5.0	50.0		40.0 ± 1.3	NR		
**Hoppe, 2016 **[50]	USA	Foley catheter	48	29.9 ± 6.0	52.1		38.9 ± 2.0	30 mL	-	①③④⑥⑧⑨⑪⑬
		Double-balloon catheter	50	30.7 ± 5.2	50.0		38.9 ± 2.1	80 mL + 80 mL		
**Sayed Ahmed, 2016 **[51]	Egypt	Foley catheter	39	25.5 ± 5.1	100		40.4 ± 2.4	50 mL	-	①②⑩
		Double-balloon catheter	39	25.7 ± 4.8	100		40.6 ± 2.4	80 mL + 80 mL		
**ten Eikelder, 2016 **[52]	Netherlands	Oral misoprostol	924	31.7 ± 5.2	66.0		39.5 ± 2.1	-	50 mcg every 4 h up to 3 doses per day up to 4 days	①②⑤⑥⑦⑧⑩⑪⑫⑬
		Foley catheter	921	31.4 ± 5.9	64.7		39.6 ± 2.1	30 mL	-	
**Yenuberi, 2016 **[53]	India	Vaginal misoprostol	380	25.0 ± 4.2	69.2		39.9 ± 1.0	-	25 mcg every 4 h up to 3 doses	①②③⑤⑥⑧⑨⑪⑬
		Oral misoprostol	383	25.5 ± 3.8	71.0		39.7 ± 1.1		50 mcg for the first dose and then 100mcg every 4 h up to 3 doses totally	
**Somirathne, 2017 **[54]	Sri Lanka	Foley catheter	89	28.8 ± 4.9	49.4		> 40.9	60 mL	-	①②③⑤⑦⑧⑩⑬
		Oral misoprostol	91	28.6 ± 5.5	50.5		> 40.9	-	50 mcg every 4 h up to 3 doses	
**Leigh, 2018 **[55]	India and UK	Foley catheter	300	24.0 ± 3.5	82.3		38.2 ± 2.2	30 mL	-	①③⑤⑥⑦⑩⑪⑫⑬
		Oral misoprostol	302	23.7 ± 3.1	78.1		38.1 ± 2.1	-	25 mcg every 2 h up to 12 doses	
**Abdi, 2021**[56]	Iran	Vaginal misoprostol	60	27.4 ± 5.4	100		42.4 ± 2.1	-	25 mcg once	①⑥⑧⑬
		Foley catheter	60	29.5 ± 6.2	100		42.8 ± 4.7	30 mL	-	
**Digusto, 2021 **[10]	France	Double-balloon catheter	607	31.1 ± 5.2	66.1		41.0 ~ 42.0	80 mL + 80 mL	-	①②⑤⑥⑦⑩⑪⑬
		Dinoprostone	609	31.3 ± 5.1	65.8		41.0 ~ 42.0	-		
**Slot, 2021**[57]	Israel	Foley catheter	94	27.8 ± 5.1	53.2		39.8 ± 1.9	40 mL	-	①④
		Double-balloon catheter	86	27.7 ± 5.8	52.3		39.9 ± 1.4	80 mL + 80 mL		

*mcg* microgram, *mL* milliliter, *PO* Per orals, *PV* Per vagina

①cesarean delivery rate; ②time from intervention-to-birth; ③achieving vaginal delivery within 24 h; ④Bishop score increment; ⑤uterine hyperstimulation with fetal heart rate changes; ⑥oxytocin augmentation; ⑦instrumental delivery; ⑧meconium-stained amniotic fluid; ⑨Chorioamnionitis; ⑩postpartum hemorrhage; ⑪Apgar score < 7 in 5 min; ⑫Apgar score < 7 in 1 min; ⑬neonatal intensive care unit admission

### Primary outcomes

**Fig. 2:**
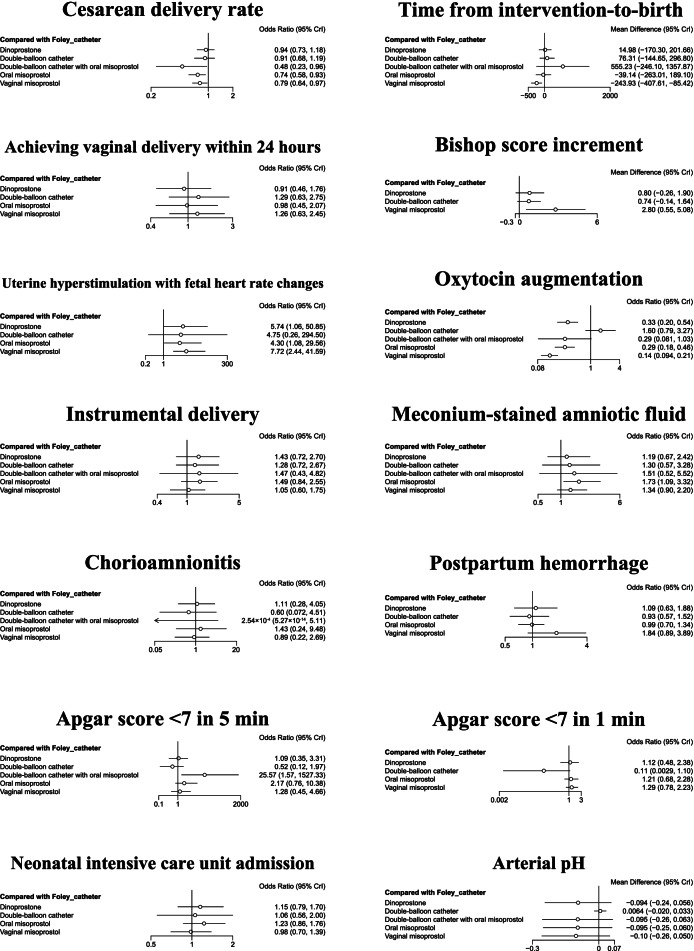
Forest plots of network meta-analysis of all trials for primary and secondary outcomes

The cesarean delivery rate in patients who underwent cervical ripening with a double-balloon catheter and oral misoprostol, oral misoprostol, and vaginal misoprostol were significantly lower than a Foley catheter (OR = 0.48, 95% CI: 0.23–0.96; OR = 0.74, 95% CI: 0.58–0.93; and OR = 0.79, 95% CI: 0.64–0.97, respectively; all *P* < 0.05; Fig. 2, Supplemental Table S3). The time from intervention-to-birth of vaginal misoprostol was significantly shorter than the other five interventions (Fig. 2, Supplemental Table S4).

### Secondary outcomes

All of the head-to-head comparisons are shown in Supplemental Table S5–S16. Compared to a Foley catheter, vaginal misoprostol resulted in a higher incremental change in the Bishop score (MD = 2.80, 95% CI: 0.55–5.08) and lower rate of oxytocin augmentation (OR = 0.14, 95% CI: 0.094–0.21), but a higher risk of uterine hyperstimulation with fetal heart rate changes (OR = 7.72, 95% CI: 2.44–41.59).

Compared to a Foley catheter, oral misoprostol had a lower rate of oxytocin augmentation (OR = 0.29, 95% CI: 0.18–0.46), but a higher risk of uterine hyperstimulation with fetal heart rate changes (OR = 4.30, 95% CI: 1.08–29.56) and a higher rate of meconium-stained amniotic fluid (OR = 1.73, 95% CI: 1.09–3.32).

Compared to a Foley catheter, a double-balloon catheter with or without oral misoprostol had similar outcomes, including uterine hyperstimulation with fetal heart rate changes (OR = 4.75, 95% CI: 0.26–294.50).

No difference in achieving vaginal delivery within 24 h, instrumental delivery, chorioamnionitis, postpartum hemorrhage, neonatal intensive care unit admission, and arterial pH among these interventions were revealed (Supplemental Tables S5, S9, S11, S12, S15, and S16).

### Network geometry, inconsistency, certainty of evidence, and publication bias

Network geometry is shown in Supplemental Figure S3. The evaluation of inconsistencies for all outcomes are presented in Supplemental Figures S4-S16. We noted a significance level (*P* > 0.05) for most cases, which indicated that inconsistency was not sufficient to influence the conclusion of this NMA. According to the SUCRA value, ranking of all interventions was done (Fig. 3). Finally, we used the GRADE system to evaluate the certainty of evidence (Table 2). No significant asymmetry was demonstrated in the funnel plot of major primary and secondary outcomes (Supplemental Figures S17 and S18). The results of Begg’s and Egger’s tests are shown in Supplemental Table S17.

**Fig. 3:**
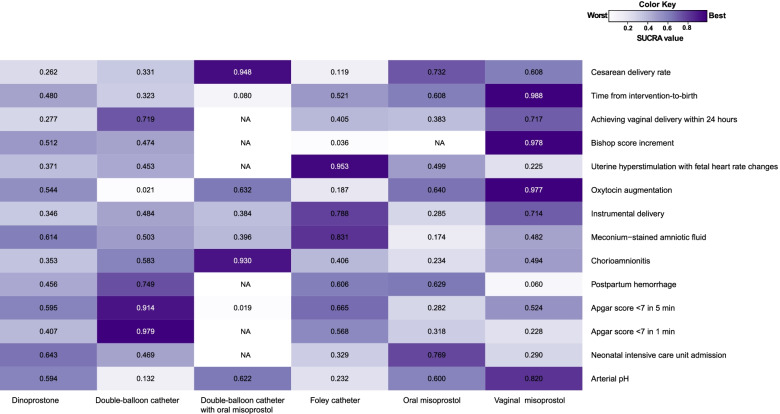
Heat maps of cervical ripening interventions for 14 Outcomes. Each column represents a cervical ripening intervention, and each row represents an outcome. Each box is colored according to the SUCRA value of the corresponding intervention and outcome. The color scale consists of values that represent SUCRA which range from 0 (white, indicating a treatment is always last) to 1 (purple, indicating a treatment is always first). Uncolored boxes labeled “NA” show that the underlying treatment was not included for that particular outcome. The values in each box represent the SUCRA value of the corresponding treatment and outcome. *NA, not applicable; SUCRA: surface under the cumulative ranking curve*

**Table 2:** Summary of the results of NMA and GRADE quality score assessment for the outcomes

Outcome	Study number	Participants number	Effect estimates (95% CI)	Conclusion	GRADE Quality score
**Cesarean delivery rate**	47	11,215	Double-balloon catheter with oral misoprostol vs. Foley catheter: OR = 0.48, 95% CI: 0.23–0.96; Oral misoprostol vs. Foley catheter: OR = 0.74, 95% CI: 0.58–0.93; Vaginal misoprostol vs. Foley catheter: OR = 0.79, 95% CI: 0.64–0.97	Double-balloon catheter with oral misoprostol, oral misoprostol, and vaginal misoprostol superior to Foley catheter	Moderate^a^
**Time from intervention-to-birth (min)**	31	7956	Vaginal misoprostol vs. double-balloon catheter with oral misoprostol: MD = -800.17, 95% CI: -1597.71–-3.01; Vaginal misoprostol vs. double-balloon catheter: MD = -320.31, 95% CI: -568.84–-74.77; Vaginal misoprostol vs. oral misoprostol: MD = -204.68, 95% CI: -414.34–-4.16; Vaginal misoprostol vs. Foley catheter: MD = -243.93, 95% CI: -407.61–-85.42; Vaginal misoprostol vs. dinoprostone: MD = -259.09, 95% CI: -450.10–-74.08;	Vaginal misoprostol superior to double-balloon catheter with oral misoprostol, double-balloon catheter, oral misoprostol, Foley catheter, and dinoprostone	Moderate^a^
**Achieving vaginal delivery within 24 h**	22	5154	More details in Supplemental Table S5	No difference among these interventions	Low^ab^
**Bishop score increment**	8	1533	Vaginal misoprostol vs. Foley catheter: MD = 2.80, 95% CI: 0.55–5.08;	Vaginal misoprostol superior to Foley catheter	Moderate^a^
**Uterine hyperstimulation with fetal heart rate changes**	27	7673	Vaginal misoprostol vs. Foley catheter: OR = 7.72, 95% CI: 2.44–41.59; Oral misoprostol vs. Foley catheter: OR = 4.30, 95% CI: 1.08–29.56; Dinoprostone vs. Foley catheter: OR = 5.74, 95% CI: 1.06–50.85	Vaginal misoprostol, oral misoprostol, and dinoprostone inferior to Foley catheter	Moderate^a^
**Oxytocin augmentation**	36	9536	Vaginal misoprostol vs. Foley catheter: OR = 0.14, 95% CI: 0.09–0.21; Oral misoprostol vs. Foley catheter: OR = 0.29, 95% CI: 0.18–0.46; Dinoprostone vs. Foley catheter: OR = 0.33, 95% CI: 0.20–0.54; Vaginal misoprostol vs. double-balloon catheter: OR = 0.09, 95% CI: 0.04–0.18; Oral misoprostol vs. double-balloon catheter: OR = 0.18, 95% CI: 0.08–0.39; Double-balloon catheter with oral misoprostol vs. double-balloon catheter: OR = 0.18, 95% CI: 0.04–0.74; Dinoprostone vs. double-balloon catheter: OR = 0.21, 95% CI: 0.12–0.36; Vaginal misoprostol vs. oral misoprostol: OR = 0.49, 95% CI: 0.34–0.69; Vaginal misoprostol vs. dinoprostone: OR = 0.42, 95% CI: 0.26–0.67;	Vaginal misoprostol, oral misoprostol, and dinoprostone superior to Foley catheter; Vaginal misoprostol, oral misoprostol, double-balloon catheter with oral misoprostol, and dinoprostone superior to double-balloon catheter; Vaginal misoprostol superior to oral misoprostol and dinoprostone	Moderate^a^
**Instrumental delivery**	25	7140	More details in Supplemental Table S9	No difference among these interventions	Moderate^a^
**Meconium-stained amniotic fluid**	28	6241	Oral misoprostol vs. Foley catheter: OR = 1.73, 95% CI: 1.09–3.32;	Oral misoprostol inferior to Foley catheter	Moderate^a^
**Chorioamnionitis**	10	2410	More details in Supplemental Table S11	No difference among these interventions	Moderate^a^
**Postpartum hemorrhage**	14	5421	More details in Supplemental Table S12	No difference among these interventions	Moderate^a^
**Apgar score < 7 in 5 min**	26	8149	Vaginal misoprostol vs. double-balloon catheter with oral misoprostol: OR = 0.05, 95% CI: 0–0.93; Double-balloon catheter vs. double-balloon catheter with oral misoprostol: OR = 0.02, 95% CI: 0–0.42; Dinoprostone vs. double-balloon catheter with oral misoprostol: OR = 0.04, 95% CI: 0–0.80; Foley catheter vs. double-balloon catheter with oral misoprostol: OR = 0.04, 95% CI: 0–0.64;	Vaginal misoprostol, double-balloon catheter, dinoprostone, and Foley catheter superior to double-balloon catheter with oral misoprostol	Moderate^a^
**Apgar score < 7 in 1 min**	16	4367	Double-balloon catheter vs. dinoprostone: OR = 0.10, 95% CI: 0–0.85; Double-balloon catheter vs. vaginal misoprostol: OR = 0.08, 95% CI: 0–0.83; Double-balloon catheter vs. oral misoprostol: OR = 0.09, 95% CI: 0–0.92;	Double-balloon catheter superior to dinoprostone, vaginal misoprostol, and oral misoprostol	Moderate^a^
**Neonatal intensive care unit admission**	34	9351	More details in Supplemental Table S15	No difference among these interventions	Moderate^a^
**Arterial pH**	9	1478	More details in Supplemental Table S16	No difference among these interventions	Moderate^a^

*CI* Confidence interval, *MD* Mean difference, *OR* Odds ratio

^a^Rated down for serious imprecision;

^b^Rated down for serious inconsistency

## Discussion

This NMA provides evidence for the relative efficacy and safety of double-balloon catheters for cervical ripening. A large amount of evidence was pooled to allow us to indirectly compare the clinical efficacy and safety profile of a double-balloon catheter with a Foley catheter, misoprostol (oral/vaginal), and a controlled-release dinoprostone insert for cervical ripening and labor induction in women with unfavorable cervices during the third trimester of pregnancy. These five methods are commonly used for cervical ripening. Our analysis demonstrated that the double-balloon catheter was not superior to other methods with respect to the cesarean section rate, time from intervention-to-birth, and maternal and neonatal adverse events. The combined use of a double-balloon catheter and oral misoprostol significantly reduced the cesarean section rate compared to a Foley catheter without an increased risk of uterine hyperstimulation with fetal heart rate changes, as occurred with oral or vaginal misoprostol alone.

To ripen the cervix, a number of methods are used; however, there is little consensus regarding which method is best [58]. It has been suggested that catheter balloons were equally effective in cervical ripening as pharmacological methods, with no significant differences in mode of delivery or perinatal outcome [59]. The double-balloon catheter was specifically developed for inducing labor. The mechanism of action by which the double-balloon catheter ripens the cervix is achieved by pressure applied to the external and internal os. The vaginal balloon is used to hold the balloon in the extra-amniotic space during cervix softening and distensibility. As the ripening process continues, the device can spontaneously expel itself early [8].

Previous systematic reviews on the safety and effectiveness of double-balloon catheters have been published; however, these reviews have been limited to pairwise meta-analyses [60–63]. In contrast, NMAs provide an important method of including a large amount of direct and indirect evidence from comparisons of many different interventions. In this NMA, we did not demonstrate an advantage to the double-balloon to other single method in various primary and secondary outcomes of labor induction. When combined with oral misoprostol, the double-balloon catheter was shown to reduce the cesarean delivery rate compared with a Foley catheter. Vaginal misoprostol alone improved the outcomes of labor induction, including the cesarean section rate, time from intervention-to-birth, Bishop score increment, and oxytocin augmentation. Even though vaginal misoprostol alone appeared to be the most effective method in cervical ripening, use of vaginal misoprostol was associated with the highest incidence of uterine hyperstimulation with fetal heart rate changes. Oral misoprostol was shown to have similar efficiency and safety to vaginal misoprostol in our analysis. The resulting uterine hyperstimulation with misoprostol use is consistent with previous studies [52, 64, 65]. Interestingly, uterine hyperstimulation with fetal heart rate changes did not occur with a double-balloon catheter combined with oral misoprostol. This finding may be due to the additional cervical dilation effect of the double-balloon catheter. This effect could reduce the misoprostol dose and the risk of uterine hyperstimulation [66].

Unlike previous studies [60, 63], we did not find any difference in Bishop score improvement between double-balloon and Foley catheters. Chorioamnionitis is a major concern when double-balloon catheters are used. According to our analysis, there were no significant difference in chorioamnionitis between a double-balloon catheter and any other method. Although there was a higher proportion of 5-min Apgar scores < 7 with double-balloon catheter and oral misoprostol use, there were only a few cases and there were no differences in umbilical artery pH, thus this finding was not clinically relevant. Therefore, this NMA indicated that the combination of a double-balloon catheter with oral misoprostol may be a preferable choice in view of the reduction in the cesarean section rate and lack of significant adverse outcomes.

Our analysis evaluated the safety and efficacy of double-balloon catheters. The combined effect of a double-balloon catheter with other cervical ripening methods was also included in our study. However, we did not identify any randomized controlled trial to assess the combined effect of controlled-release dinoprostone and a double-balloon catheter, although this combination may improve the induction outcome much like the combined effect with misoprostol. The high cost of controlled-release dinoprostone and a double-balloon catheter should be the reason. We did not perform an NMA to compare the combined effect of a Foley catheter with other cervical ripening methods in the present study. Because safety and efficacy was similar between double-balloon and Foley catheters, whether a Foley catheter combined with misoprostol has the same effect needs to be confirmed. It should be noted that a Foley catheter is much less expensive than a double-balloon catheter. In fact, use of a Foley catheter is a classic mechanical method for cervical ripening and widely used in low-resource settings [55, 67]. Among developing countries where health-related costs are a major concern, a Foley catheter is recommended as a better option than other cervical ripening methods.

### Strengths

One of the strengths of our review was the application of an NMA. Our NMA was strictly confined to randomized trials and provided comprehensive comparisons between a double-balloon catheter and five other cervical ripening techniques, which increased the interpretation of the existing evidence. We calculated the probabilities of ranking cervical ripening methods using Bayesian analysis. Furthermore, to minimize potential bias due to the variation in the characteristics of the included women, we applied several restrictions for inclusion in the review. Specifically, we excluded studies that included outpatients or pregnant women who were in the second trimester. Third, only few included trials were of low quality. Moreover, our protocol was registered with PROSPERO before data abstraction commenced.

### Future directions

First, because a Foley catheter is much less expensive than a double-balloon catheter, trials aimed to compare the efficacy of “the combination of a Foley catheter with misoprostol” and “the combination of a double-balloon catheter with misoprostol” needs to be conducted. Second, compared with inpatient management, women may be able to find better psychological and social support at home. Therefore, the safety of outpatient cervical priming of a double-balloon catheter also needs to be confirmed. Third, only one trial compared a double-balloon catheter with oral misoprostol to oral misoprostol alone [11], thus additional evidence is needed.

### Limitation

The current meta-analysis had some limitations. First, to decrease the heterogeneity, we only included trials with the dinoprostone formulation that was most often used in the trials compared with a double-balloon catheter. Second, the misoprostol dose and the volume of the double-balloon or Foley catheter were variable, which may affect the credibility of the conclusion. Third, the characteristics of the participants, such as maternal age, parity, gestational age, body mass index, baseline Bishop score, and labor induction, were diverse and underlying confounders. Fourth, some of the involved trials were not double-blinded due to the nature of the intervention.

## Conclusion

The clinical outcomes were similar between a double-balloon catheter alone and other single methods. For pregnant women with intact membranes after 28 weeks gestation, vaginal misoprostol was shown to be the most effective methods for cervical ripening with respect to the cesarean delivery rate, time from intervention-to-birth, and oxytocin augmentation; however, vaginal misoprostol was associated with higher rates of uterine hyperstimulation with fetal heart rate changes. The combination of a double-balloon catheter with oral misoprostol was the best method to reduce the likelihood of delivery by cesarean section without uterine hyperstimulation with fetal heart rate changes. 

## Supplementary Information

**Additional file 1:** **TableS1.** PRISMA Network Meta-analysis Checklist. **TableS2.** Strategy of this meta-analysis. **TableS3.** Head-to-head comparisons of cesarean delivery rate. **Table S4.** Head-to-head comparisons oftime from intervention-to-birth. **TableS5.** Head-to-head comparisons of achieving vaginal delivery within 24 hours.**Table S6.** Head-to-head comparisonsof Bishop score increment. **Table S7.** Head-to-head comparisons of uterine hyperstimulation with fetal heart ratechanges. **Table S8.** Head-to-headcomparisons of oxytocin augmentation. **TableS9.** Head-to-head comparisons of instrumental delivery. **Table S10.** Head-to-head comparisons of meconium-stained amnioticfluid. **Table S11.** Head-to-headcomparisons of chorioamnionitis. **TableS12.** Head-to-head comparisons of postpartum hemorrhage. **Table S13.** Head-to-head comparisons ofApgar score <7 in 5 min. **Table S14.**Head-to-head comparisons of Apgar score <7 in 1 min. **Figure S15.** Inconsistency test of Apgar score <7 in 1 min. **Table S16.** Head-to-head comparisons ofarterial pH. **Table S17.** Assessmentof publication bias for network meta-analysis. **Figure S1.** Risk of bias summary. **Figure S2.** Risk of bias graph. **FigureS2.** Risk of bias graph. **Figure S4.** Inconsistency test of cesarean delivery rate. **Figure S5.** Inconsistency test of Time from intervention-to-birth. **Figure S6.** Inconsistency test ofachieving vaginal delivery within 24 hours. **Figure S7.** Inconsistency test of Bishop score increment. **Figure S8.** Inconsistency test ofuterine hyperstimulation with fetal heart rate changes. **Figure S9.** Inconsistency test of oxytocin augmentation. **Figure S10.** Inconsistency test ofinstrumental delivery. **Figure S11.** Inconsistency test of meconium-stained amniotic fluid. **Figure S12.** Inconsistency test of chorioamnionitis. **Figure S13.** Inconsistency test ofpostpartum hemorrhage. **Figure S14.** Inconsistency test of Apgar score <7 in 5 min. **Figure S15.** Inconsistency test of Apgar score <7 in 1 min. **Figure S16.** Inconsistency test ofneonatal intensive care unit admission. **Figure S17.** Funnel plot of primary outcomes. **Figure S18.** Funnel plot of secondary outcomes.

## Data Availability

The datasets used and/or analyzed during the current study are available from the corresponding author on reasonable request.

## Abbreviations

CI: Confidence interval
MD: Mean difference
NMA: Network meta-analysis
OR: Odds ratio
RCT: Randomized controlled trial
SUCRA: Surface under the cumulative ranking curve
