# Supplementary material for: Safety and efficacy of double-balloon catheter for cervical ripening: a Bayesian network meta-analysis of randomized controlled trials
Source: BMC Pregnancy Childbirth. 2022 Sep 6;22:688. doi: 10.1186/s12884-022-04988-2 (PMC9450369; doi:10.1186/s12884-022-04988-2)

**Safety and efficacy of double-balloon catheter for cervical ripening: a Bayesian network meta-analysis of randomized controlled trials**

Content

[Table S1. PRISMA Network Meta-analysis Checklist 2](#_Toc106450208)

[Table S2. Strategy of this meta-analysis 3](#_Toc106450209)

[Table S3. Head-to-head comparisons of cesarean delivery rate 4](#_Toc106450210)

[Table S4. Head-to-head comparisons of time from intervention-to-birth 4](#_Toc106450211)

[Table S5. Head-to-head comparisons of achieving vaginal delivery within 24 hours 4](#_Toc106450212)

[Table S6. Head-to-head comparisons of Bishop score increment 4](#_Toc106450213)

[Table S7. Head-to-head comparisons of uterine hyperstimulation with fetal heart rate changes 4](#_Toc106450214)

[Table S8. Head-to-head comparisons of oxytocin augmentation 5](#_Toc106450215)

[Table S9. Head-to-head comparisons of instrumental delivery 5](#_Toc106450216)

[Table S10. Head-to-head comparisons of meconium-stained amniotic fluid 5](#_Toc106450217)

[Table S11. Head-to-head comparisons of chorioamnionitis 5](#_Toc106450218)

[Table S12. Head-to-head comparisons of postpartum hemorrhage 5](#_Toc106450219)

[Table S13. Head-to-head comparisons of Apgar score <7 in 5 min 6](#_Toc106450220)

[Table S14. Head-to-head comparisons of Apgar score <7 in 1 min 6](#_Toc106450221)

[Table S15. Head-to-head comparisons of neonatal intensive care unit admission 6](#_Toc106450222)

[Table S16. Head-to-head comparisons of arterial pH 6](#_Toc106450223)

[Table S17. Assessment of publication bias for network meta-analysis 7](#_Toc106450224)

[Figure S1. Risk of bias summary. 8](#_Toc106450225)

[Figure S2. Risk of bias graph. 9](#_Toc106450226)

[Figure S3. Network geometry 10](#_Toc106450227)

[Figure S4. Inconsistency test of cesarean delivery rate 11](#_Toc106450228)

[Figure S5. Inconsistency test of Time from intervention-to-birth 11](#_Toc106450229)

[Figure S6. Inconsistency test of achieving vaginal delivery within 24 hours 12](#_Toc106450230)

[Figure S7. Inconsistency test of Bishop score increment 12](#_Toc106450231)

[Figure S8. Inconsistency test of uterine hyperstimulation with fetal heart rate changes 13](#_Toc106450232)

[Figure S9. Inconsistency test of oxytocin augmentation 13](#_Toc106450233)

[Figure S10. Inconsistency test of instrumental delivery 14](#_Toc106450234)

[Figure S11. Inconsistency test of meconium-stained amniotic fluid 14](#_Toc106450235)

[Figure S12. Inconsistency test of chorioamnionitis 15](#_Toc106450236)

[Figure S13. Inconsistency test of postpartum hemorrhage 15](#_Toc106450237)

[Figure S14. Inconsistency test of Apgar score <7 in 5 min 16](#_Toc106450238)

[Figure S15. Inconsistency test of Apgar score <7 in 1 min 16](#_Toc106450239)

[Figure S16. Inconsistency test of neonatal intensive care unit admission 17](#_Toc106450240)

[Figure S17. Funnel plot of primary outcomes 18](#_Toc106450241)

[Figure S18. Funnel plot of secondary outcomes. 19](#_Toc106450242)

# Table S1. PRISMA Network Meta-analysis Checklist

| **TITLE** |  |  | **PAGE** |
| --- | --- | --- | --- |
| Title | 1 | Identify the report as a systematic review *incorporating a network meta-analysis (or related form of meta-analysis).* | **1** |
| **ABSTRACT** |  |  |  |
| Structured summary | 2 | Provide a structured summary including, as applicable:  **Background:** main objectives  **Methods:** data sources; study eligibility criteria, participants, and interventions; study appraisal; and *synthesis methods, such as network meta-analysis.*  **Results:** number of studies and participants identified; summary estimates with corresponding confidence/credible intervals; *treatment rankings may also be discussed. Authors may choose to summarize pairwise comparisons against a chosen treatment included in their analyses for brevity.*  **Discussion/Conclusions:** limitations; conclusions and implications of findings.  **Other:** primary source of funding; systematic review registration number with registry name. | **2** |
| **INTRODUCTION** |  |  |  |
| Rationale | 3 | Describe the rationale for the review in the context of what is already known*, including mention of why a network meta-analysis has been conducted.* | **4** |
| Objectives | 4 | Provide an explicit statement of questions being addressed, with reference to participants, interventions, comparisons, outcomes, and study design (PICOS). | **4** |
| **METHODS** |  |  |  |
| Protocol and registration | 5 | Indicate whether a review protocol exists and if and where it can be accessed (e.g., Web address); and, if available, provide registration information, including registration number. | **5-6** |
| Eligibility criteria | 6 | Specify study characteristics (e.g., PICOS, length of follow-up) and report characteristics (e.g., years considered, language, publication status) used as criteria for eligibility, giving rationale. *Clearly describe eligible treatments included in the treatment network, and note whether any have been clustered or merged into the same node (with justification).* | **6** |
| Information sources | 7 | Describe all information sources (e.g., databases with dates of coverage, contact with study authors to identify additional studies) in the search and date last searched. | 6 |
| Search | 8 | Present full electronic search strategy for at least one database, including any limits used, such that it could be repeated. | 6 |
| Study selection | 9 | State the process for selecting studies (i.e., screening, eligibility, included in systematic review, and, if applicable, included in the meta-analysis). | **6** |
| Data collection process | 10 | Describe method of data extraction from reports (e.g., piloted forms, independently, in duplicate) and any processes for obtaining and confirming data from investigators. | **6** |
| Data items | 11 | List and define all variables for which data were sought (e.g., PICOS, funding sources) and any assumptions and simplifications made. | **6** |
| **Geometry of the network** | **S1** | Describe methods used to explore the geometry of the treatment network under study and potential biases related to it. This should include how the evidence base has been graphically summarized for presentation, and what characteristics were compiled and used to describe the evidence base to readers. | **Supplemental file** |
| Risk of bias within individual studies | 12 | Describe methods used for assessing risk of bias of individual studies (including specification of whether this was done at the study or outcome level), and how this information is to be used in any data synthesis. | **7** |
| Summary measures | 13 | State the principal summary measures (e.g., risk ratio, difference in means). *Also describe the use of additional summary measures assessed, such as treatment rankings and surface under the cumulative ranking curve (SUCRA) values, as well as modified approaches used to present summary findings from meta-analyses.* | **7** |
| Planned methods of analysis | 14 | Describe the methods of handling data and combining results of studies for each network meta-analysis. This should include, but not be limited to:   - *Handling of multi-arm trials;* - *Selection of variance structure;* - *Selection of prior distributions in Bayesian analyses; and* - *Assessment of model fit.* | **7** |
| **Assessment of Inconsistency** | **S2** | Describe the statistical methods used to evaluate the agreement of direct and indirect evidence in the treatment network(s) studied. Describe efforts taken to address its presence when found. | **8** |
| Risk of bias across studies | 15 | Specify any assessment of risk of bias that may affect the cumulative evidence (e.g., publication bias, selective reporting within studies). | **8** |
| Additional analyses | 16 | Describe methods of additional analyses if done, indicating which were pre-specified. This may include, but not be limited to, the following:   - Sensitivity or subgroup analyses; - Meta-regression analyses; - *Alternative formulations of the treatment network; and* - *Use of alternative prior distributions for Bayesian analyses (if applicable).* | **NR** |
| **RESULTS†** |  |  |  |
| Study selection | 17 | Give numbers of studies screened, assessed for eligibility, and included in the review, with reasons for exclusions at each stage, ideally with a flow diagram. | **8** |
| **Presentation of network structure** | **S3** | Provide a network graph of the included studies to enable visualization of the geometry of the treatment network. | **Supplemental file** |
| **Summary of network geometry** | **S4** | Provide a brief overview of characteristics of the treatment network. This may include commentary on the abundance of trials and randomized patients for the different interventions and pairwise comparisons in the network, gaps of evidence in the treatment network, and potential biases reflected by the network structure. | **8** |
| Study characteristics | 18 | For each study, present characteristics for which data were extracted (e.g., study size, PICOS, follow-up period) and provide the citations. | **8** |
| Risk of bias within studies | 19 | Present data on risk of bias of each study and, if available, any outcome level assessment. | **Supplemental file** |
| Results of individual studies | 20 | For all outcomes considered (benefits or harms), present, for each study: 1) simple summary data for each intervention group, and 2) effect estimates and confidence intervals. *Modified approaches may be needed to deal with information from larger networks.* | **9-10** |
| Synthesis of results | 21 | Present results of each meta-analysis done, including confidence/credible intervals. *In larger networks, authors may focus on comparisons versus a particular comparator (e.g. placebo or standard care), with full findings presented in an appendix. League tables and forest plots may be considered to summarize pairwise comparisons.* If additional summary measures were explored (such as treatment rankings), these should also be presented. | **9-10** |
| **Exploration for inconsistency** | **S5** |  | **Supplemental file** |
| Risk of bias across studies | 22 | Present results of any assessment of risk of bias across studies for the evidence base being studied. | **10** |
| Results of additional analyses | 23 | Give results of additional analyses, if done (e.g., sensitivity or subgroup analyses, meta-regression analyses*, alternative network geometries studied, alternative choice of prior distributions for Bayesian analyses,* and so forth). | **NR** |
| **DISCUSSION** |  |  |  |
| Summary of evidence | 24 | Summarize the main findings, including the strength of evidence for each main outcome; consider their relevance to key groups (e.g., healthcare providers, users, and policy-makers). | **10-13** |
| Limitations | 25 | Discuss limitations at study and outcome level (e.g., risk of bias), and at review level (e.g., incomplete retrieval of identified research, reporting bias). *Comment on the validity of the assumptions, such as transitivity and consistency. Comment on any concerns regarding network geometry (e.g., avoidance of certain comparisons).* | **13** |
| Conclusions | 26 | Provide a general interpretation of the results in the context of other evidence, and implications for future research. | **13-14** |
| **FUNDING** |  |  |  |
| Funding | 27 | Describe sources of funding for the systematic review and other support (e.g., supply of data); role of funders for the systematic review. This should also include information regarding whether funding has been received from manufacturers of treatments in the network and/or whether some of the authors are content experts with professional conflicts of interest that could affect use of treatments in the network. | **14** |

PICOS = population, intervention, comparators, outcomes, study design. † Authors may wish to plan for use of appendices to present all relevant information in full detail for items in this section.

# Table S2. Strategy of this meta-analysis

|  | Strategy |
| --- | --- |
| PubMed | #1: randomized controlled trial[Publication Type] OR controlled clinical trial[Publication Type] OR "Randomized Controlled Trials as Topic"[Mesh] OR randomized[Title/Abstract] OR random[Title/Abstract] OR randomly[Title/Abstract] OR controlled[Title/Abstract] OR trial[Title/Abstract] OR placebo[Title/Abstract] OR groups[Title/Abstract]  #2: "Cervical Ripening"[Mesh] OR cervical ripening*[Title/Abstract] OR "Labor, Induced"[Mesh] OR labor induc*[Title/Abstract] OR labour induc*[Title/Abstract] OR cervical ripen[Title/Abstract] OR cervical priming[Title/Abstract] OR mechanical dilation* [Title/Abstract] OR mechanical dilatation*[Title/Abstract] OR mechanical method* [Title/Abstract]  #3: ("Prostaglandins"[Mesh] OR Prostaglandin[Title/Abstract] OR Prostanoid*[Title/Abstract]) OR ("Dinoprostone"[Mesh] OR Dinoprostone[Title/Abstract] OR PGE2*[Title/Abstract] OR Prostaglandin E2*[Title/Abstract] OR Prepidil Gel[Title/Abstract] OR Prostenon[Title/Abstract] OR PGE2 alpha[Title/Abstract] OR Propess[Title/Abstract]) OR ("Misoprostol"[Mesh] OR Misoprostol[Title/Abstract] OR Cytotec[Title/Abstract] OR SC-30249[Title/Abstract] OR PGE1[Title/Abstract]) OR ("Catheters"[Mesh] OR Foley[Title/Abstract] OR catheter*[Title/Abstract] OR balloon[Title/Abstract] OR "Catheterization"[Mesh] OR catheterization[Title/Abstract])  #4: #1 AND #2 AND #3 |
| MEDLINE | #1: (randomized controlled trial.pt OR controlled clinical trial.pt OR randomized.mp. OR placebo.mp. OR randomly.mp. OR trial.ti. OR Clinical Trials as Topic/) NOT (animals.sh. NOT humans.sh.)  #2: (Cervical Ripening OR Labor, Induced).sh. OR (cervical ripening* OR labor induc* OR labour induc* OR cervical ripen OR cervical priming OR mechanical dilation* OR mechanical dilatation* OR mechanical method*).mp.  #3: (Prostaglandins OR Dinoprostone OR Misoprostol OR Catheters OR Catheterization).sh. OR (Prostaglandin OR Prostanoid* OR Dinoprostone OR PGE2* OR Prostaglandin E2* OR Prepidil Gel OR Prostenon OR PGE2 alpha OR Propess OR Misoprostol OR Cytotec OR SC-30249 OR PGE1 OR Foley OR catheter* OR balloon OR catheterization).mp.  #4: #1 AND #2 AND #3 |
| Embase | #1: 'random':ab,ti OR 'placebo':ab,ti OR 'double-blind':ab,ti OR 'randomized':ab,ti OR 'controlled':ab,ti OR 'group':ab,ti OR 'trial':ab,ti  #2: 'Cervical Ripening'/exp OR 'cervical ripening*':ab,ti OR 'Labor, Induced'/exp OR 'labor induc*':ab,ti OR 'labour induc*':ab,ti OR 'cervical ripen':ab,ti OR 'cervical priming':ab,ti OR 'mechanical dilation*':ab,ti OR 'mechanical dilatation*':ab,ti OR 'mechanical method*':ab,ti  #3: 'Prostaglandins'/exp OR 'Prostaglandin':ab,ti OR 'Prostanoid*':ab,ti OR 'Dinoprostone'/exp OR 'Dinoprostone':ab,ti OR 'PGE2*':ab,ti OR 'Prostaglandin E2*':ab,ti OR 'Prepidil Gel':ab,ti OR 'Prostenon':ab,ti OR 'PGE2 alpha':ab,ti OR 'Propess':ab,ti OR 'Misoprostol'/exp OR 'Misoprostol':ab,ti OR 'Cytotec':ab,ti OR 'SC-30249':ab,ti OR 'PGE1':ab,ti OR 'Catheters'/exp OR 'Foley':ab,ti OR 'catheter*':ab,ti OR 'balloon':ab,ti OR 'Catheterization'/exp OR 'catheterization':ab,ti  #4: #1 AND #2 AND #3 |

# Table S3. Head-to-head comparisons of cesarean delivery rate

| Dinoprostone | 0.98 (0.74, 1.26) | 0.51 (0.24, 1.05) | 1.07 (0.85, 1.37) | 0.79 (0.59, 1.05) | 0.84 (0.65, 1.1) |
| --- | --- | --- | --- | --- | --- |
| 1.02 (0.79, 1.35) | Double-balloon catheter | 0.53 (0.25, 1.11) | 1.1 (0.84, 1.47) | 0.81 (0.58, 1.15) | 0.87 (0.63, 1.21) |
| 1.95 (0.95, 4.09) | 1.9 (0.9, 4.04) | Double-balloon catheter with oral misoprostol | 2.08 (1.04, 4.32) | 1.55 (0.8, 3.04) | 1.65 (0.83, 3.38) |
| 0.94 (0.73, 1.18) | 0.91 (0.68, 1.19) | 0.48 (0.23, 0.96) | Foley catheter | 0.74 (0.58, 0.93) | 0.79 (0.64, 0.97) |
| 1.26 (0.95, 1.69) | 1.23 (0.87, 1.73) | 0.65 (0.33, 1.25) | 1.35 (1.08, 1.73) | Oral misoprostol | 1.07 (0.87, 1.32) |
| 1.18 (0.91, 1.53) | 1.15 (0.83, 1.58) | 0.61 (0.3, 1.2) | 1.27 (1.03, 1.56) | 0.94 (0.76, 1.15) | Vaginal misoprostol |

Data in red color means *P* < 0.05

# Table S4. Head-to-head comparisons of time from intervention-to-birth

| Dinoprostone | 61.21 (-153.87, 273.48) | 540.21 (-271.61, 1351.35) | -14.98 (-201.66, 170.3) | -54.4 (-314.6, 209.73) | -259.09 (-450.1, -74.08) |
| --- | --- | --- | --- | --- | --- |
| -61.21 (-273.48, 153.87) | Double-balloon_catheter | 478.35 (-346.22, 1304.85) | -76.31 (-296.8, 144.65) | -116.15 (-411.82, 187.74) | -320.31 (-568.84, -74.77) |
| -540.21 (-1351.35, 271.61) | -478.35 (-1304.85, 346.22) | Double-balloon catheter with oral misoprostol | -555.23 (-1357.87, 246.1) | -593.33 (-1362.76, 175.52) | -800.17 (-1597.71, -3.01) |
| 14.98 (-170.3, 201.66) | 76.31 (-144.65, 296.8) | 555.23 (-246.1, 1357.87) | Foley catheter | -39.14 (-263.01, 189.1) | -243.93 (-407.61, -85.42) |
| 54.4 (-209.73, 314.6) | 116.15 (-187.74, 411.82) | 593.33 (-175.52, 1362.76) | 39.14 (-189.1, 263.01) | Oral misoprostol | -204.68 (-414.34, -4.16) |
| 259.09 (74.08, 450.1) | 320.31 (74.77, 568.84) | 800.17 (3.01, 1597.71) | 243.93 (85.42, 407.61) | 204.68 (4.16, 414.34) | Vaginal misoprostol |

Data in red color means *P* < 0.05

# Table S5. Head-to-head comparisons of achieving vaginal delivery within 24 hours

| Dinoprostone | 1.43 (0.72, 2.94) | 1.1 (0.57, 2.17) | 1.08 (0.48, 2.41) | 1.39 (0.71, 2.69) |
| --- | --- | --- | --- | --- |
| 0.7 (0.34, 1.38) | Double-balloon catheter | 0.77 (0.36, 1.59) | 0.76 (0.28, 1.93) | 0.97 (0.4, 2.25) |
| 0.91 (0.46, 1.76) | 1.29 (0.63, 2.75) | Foley catheter | 0.98 (0.45, 2.07) | 1.26 (0.63, 2.45) |
| 0.93 (0.41, 2.09) | 1.32 (0.52, 3.52) | 1.02 (0.48, 2.2) | Oral misoprostol | 1.29 (0.67, 2.47) |
| 0.72 (0.37, 1.41) | 1.03 (0.44, 2.5) | 0.79 (0.41, 1.58) | 0.78 (0.4, 1.49) | Vaginal misoprostol |

# Table S6. Head-to-head comparisons of Bishop score increment

| Dinoprostone | -0.06 (-0.95, 0.81) | -0.8 (-1.9, 0.26) | 2 (-0.01, 4) |
| --- | --- | --- | --- |
| 0.06 (-0.81, 0.95) | Double-balloon catheter | -0.74 (-1.64, 0.14) | 2.06 (-0.13, 4.25) |
| 0.8 (-0.26, 1.9) | 0.74 (-0.14, 1.64) | Foley catheter | 2.8 (0.55, 5.08) |
| -2 (-4, 0.01) | -2.06 (-4.25, 0.13) | -2.8 (-5.08, -0.55) | Vaginal misoprostol |

Data in red color means *P* < 0.05

# Table S7. Head-to-head comparisons of uterine hyperstimulation with fetal heart rate changes

| Dinoprostone | 0.82 (0.07, 20.81) | 0.17 (0.02, 0.95) | 0.75 (0.1, 6.28) | 1.34 (0.26, 7.81) |
| --- | --- | --- | --- | --- |
| 1.22 (0.05, 13.38) | Double-balloon catheter | 0.21 (0, 3.8) | 0.91 (0.02, 20.82) | 1.63 (0.04, 30.12) |
| 5.74 (1.06, 50.85) | 4.75 (0.26, 294.53) | Foley catheter | 4.30 (1.08, 29.56) | 7.72 (2.44, 41.59) |
| 1.33 (0.16, 10.15) | 1.1 (0.05, 50.77) | 0.23 (0.03, 0.92) | Oral misoprostol | 1.78 (0.49, 6.61) |
| 0.75 (0.13, 3.92) | 0.61 (0.03, 23.14) | 0.13 (0.02, 0.41) | 0.56 (0.15, 2.04) | Vaginal misoprostol |

Data in red color means *P* < 0.05

# Table S8. Head-to-head comparisons of oxytocin augmentation

| Dinoprostone | 4.78 (2.76, 8.4) | 0.88 (0.24, 3.19) | 2.99 (1.85, 4.89) | 0.87 (0.5, 1.51) | 0.42 (0.26, 0.67) |
| --- | --- | --- | --- | --- | --- |
| 0.21 (0.12, 0.36) | Double-balloon catheter | 0.18 (0.04, 0.74) | 0.62 (0.31, 1.27) | 0.18 (0.08, 0.39) | 0.09 (0.04, 0.18) |
| 1.14 (0.31, 4.2) | 5.46 (1.36, 22.42) | Double-balloon catheter with oral misoprostol | 3.41 (0.97, 12.24) | 1 (0.31, 3.24) | 0.48 (0.14, 1.65) |
| 0.33 (0.20, 0.54) | 1.6 (0.79, 3.27) | 0.29 (0.08, 1.03) | Foley catheter | 0.29 (0.18, 0.46) | 0.14 (0.09, 0.21) |
| 1.15 (0.66, 1.99) | 5.48 (2.57, 11.86) | 1 (0.31, 3.24) | 3.42 (2.17, 5.5) | Oral misoprostol | 0.49 (0.34, 0.69) |
| 2.36 (1.48, 3.82) | 11.29 (5.6, 23.41) | 2.06 (0.61, 7.09) | 7.04 (4.81, 10.6) | 2.06 (1.44, 2.98) | Vaginal misoprostol |

Data in red color means *P* < 0.05

# Table S9. Head-to-head comparisons of instrumental delivery

| Dinoprostone | 0.88 (0.56, 1.85) | 1.02 (0.26, 4.13) | 0.7 (0.37, 1.39) | 1.04 (0.45, 2.5) | 0.73 (0.32, 1.7) |
| --- | --- | --- | --- | --- | --- |
| 1.13 (0.54, 1.79) | Double-balloon catheter | 1.15 (0.26, 4.11) | 0.78 (0.37, 1.39) | 1.17 (0.44, 2.48) | 0.82 (0.31, 1.73) |
| 0.98 (0.24, 3.82) | 0.87 (0.24, 3.83) | Double-balloon catheter with oral misoprostol | 0.68 (0.21, 2.32) | 1.01 (0.35, 2.97) | 0.72 (0.22, 2.28) |
| 1.43 (0.72, 2.7) | 1.28 (0.72, 2.67) | 1.47 (0.43, 4.82) | Foley catheter | 1.49 (0.84, 2.55) | 1.05 (0.59, 1.75) |
| 0.96 (0.4, 2.23) | 0.86 (0.4, 2.25) | 0.99 (0.34, 2.88) | 0.67 (0.39, 1.19) | Oral misoprostol | 0.71 (0.43, 1.1) |
| 1.36 (0.59, 3.17) | 1.21 (0.58, 3.21) | 1.4 (0.44, 4.64) | 0.95 (0.57, 1.68) | 1.41 (0.91, 2.31) | Vaginal misoprostol |

# Table S10. Head-to-head comparisons of meconium-stained amniotic fluid

| Dinoprostone | 1.09 (0.49, 2.32) | 1.27 (0.38, 4.57) | 0.84 (0.41, 1.49) | 1.45 (0.75, 2.95) | 1.12 (0.62, 1.99) |
| --- | --- | --- | --- | --- | --- |
| 0.91 (0.43, 2.04) | Double-balloon catheter | 1.16 (0.3, 5.03) | 0.77 (0.31, 1.75) | 1.33 (0.54, 3.58) | 1.03 (0.43, 2.49) |
| 0.79 (0.22, 2.66) | 0.86 (0.2, 3.33) | Double-balloon catheter with oral misoprostol | 0.66 (0.18, 1.91) | 1.14 (0.4, 3.22) | 0.88 (0.27, 2.57) |
| 1.19 (0.67, 2.42) | 1.3 (0.57, 3.27) | 1.5 (0.52, 5.52) | Foley catheter | 1.73 (1.09, 3.32) | 1.34 (0.9, 2.2) |
| 0.69 (0.34, 1.33) | 0.75 (0.28, 1.84) | 0.88 (0.31, 2.49) | 0.58 (0.3, 0.92) | Oral misoprostol | 0.77 (0.49, 1.11) |
| 0.89 (0.5, 1.62) | 0.97 (0.4, 2.33) | 1.13 (0.39, 3.66) | 0.75 (0.45, 1.11) | 1.29 (0.9, 2.02) | Vaginal misoprostol |

Data in red color means *P* < 0.05

# Table S11. Head-to-head comparisons of chorioamnionitis

| Dinoprostone | 0.54 (0.05, 6.26) | 0 (0, 4.65) | 0.91 (0.25, 3.57) | 1.29 (0.23, 8.83) | 0.82 (0.21, 2.43) |
| --- | --- | --- | --- | --- | --- |
| 1.85 (0.16, 21.6) | Double-balloon catheter | 0 (0, 10.85) | 1.67 (0.22, 13.81) | 2.4 (0.16, 40.59) | 1.48 (0.12, 15.03) |
| 4255.93 (0.22, 2.14*10^13^) | 2394.09 (0.09, 1.25*10^13^) | Double-balloon catheter with oral misoprostol | 3930.06 (0.2, 1.90*10^13^) | 5549.19 (0.36, 2.79*10^13^) | 3297.42 (0.18, 1.69*10^13^) |
| 1.1 (0.28, 4.05) | 0.6 (0.07, 4.51) | 0 (0, 5.11) | Foley catheter | 1.43 (0.24, 9.48) | 0.89 (0.22, 2.69) |
| 0.78 (0.11, 4.41) | 0.42 (0.02, 6.15) | 0 (0, 2.8) | 0.7 (0.11, 4.16) | Oral misoprostol | 0.63 (0.12, 2.07) |
| 1.23 (0.41, 4.85) | 0.68 (0.07, 8.26) | 0 (0, 5.45) | 1.12 (0.37, 4.56) | 1.58 (0.48, 8.11) | Vaginal misoprostol |

# Table S12. Head-to-head comparisons of postpartum hemorrhage

| Dinoprostone | 0.85 (0.59, 1.23) | 0.92 (0.53, 1.58) | 0.91 (0.49, 1.67) | 1.7 (0.76, 3.83) |
| --- | --- | --- | --- | --- |
| 1.18 (0.81, 1.7) | Double-balloon catheter | 1.08 (0.66, 1.77) | 1.07 (0.59, 1.88) | 2 (0.88, 4.49) |
| 1.09 (0.63, 1.88) | 0.93 (0.57, 1.52) | Foley catheter | 0.99 (0.7, 1.34) | 1.84 (0.89, 3.89) |
| 1.1 (0.6, 2.06) | 0.94 (0.53, 1.7) | 1.01 (0.74, 1.43) | Oral misoprostol | 1.87 (0.9, 4.05) |
| 0.59 (0.26, 1.31) | 0.5 (0.22, 1.14) | 0.54 (0.26, 1.12) | 0.53 (0.25, 1.11) | Vaginal misoprostol |

# Table S13. Head-to-head comparisons of Apgar score <7 in 5 min

| Dinoprostone | 0.48 (0.13, 1.6) | 23.61 (1.25, 1564.6) | 0.92 (0.3, 2.88) | 2.02 (0.5, 13.17) | 1.19 (0.35, 5.22) |
| --- | --- | --- | --- | --- | --- |
| 2.08 (0.63, 7.9) | Double-balloon catheter | 49.39 (2.36, 3872.03) | 1.92 (0.51, 8.55) | 4.24 (0.85, 37.92) | 2.47 (0.55, 16.75) |
| 0.04 (0, 0.8) | 0.02 (0, 0.42) | Double-balloon catheter with oral misoprostol | 0.04 (0, 0.64) | 0.09 (0, 1.24) | 0.05 (0, 0.93) |
| 1.09 (0.35, 3.31) | 0.52 (0.12, 1.97) | 25.57 (1.57, 1527.33) | Foley catheter | 2.17 (0.76, 10.38) | 1.28 (0.45, 4.66) |
| 0.49 (0.08, 1.99) | 0.24 (0.03, 1.17) | 11.2 (0.8, 508.47) | 0.46 (0.1, 1.32) | Oral misoprostol | 0.58 (0.14, 1.96) |
| 0.84 (0.19, 2.88) | 0.4 (0.06, 1.83) | 19.64 (1.07, 1104.15) | 0.78 (0.21, 2.2) | 1.72 (0.51, 7.03) | Vaginal misoprostol |

Data in red color means *P* < 0.05

# Table S14. Head-to-head comparisons of Apgar score <7 in 1 min

| Dinoprostone | 0.1 (0, 0.85) | 0.89 (0.42, 2.1) | 1.08 (0.47, 2.86) | 1.15 (0.58, 2.6) |
| --- | --- | --- | --- | --- |
| 10.34 (1.18, 365.39) | Double-balloon catheter | 9.4 (0.91, 348.69) | 11.47 (1.09, 458.17) | 12.18 (1.2, 448.64) |
| 1.12 (0.48, 2.37) | 0.11 (0, 1.1) | Foley catheter | 1.21 (0.68, 2.28) | 1.29 (0.78, 2.23) |
| 0.93 (0.35, 2.11) | 0.09 (0, 0.92) | 0.83 (0.44, 1.47) | Oral misoprostol | 1.06 (0.6, 1.87) |
| 0.87 (0.38, 1.72) | 0.08 (0, 0.83) | 0.78 (0.45, 1.28) | 0.94 (0.53, 1.67) | Vaginal misoprostol |

Data in red color means *P* < 0.05

# Table S15. Head-to-head comparisons of neonatal intensive care unit admission

| Dinoprostone | 0.92 (0.53, 1.6) | 0.87 (0.59, 1.27) | 1.07 (0.69, 1.66) | 0.85 (0.58, 1.26) |
| --- | --- | --- | --- | --- |
| 1.09 (0.62, 1.89) | Double-balloon catheter | 0.95 (0.5, 1.8) | 1.16 (0.59, 2.35) | 0.93 (0.48, 1.79) |
| 1.15 (0.79, 1.7) | 1.06 (0.56, 2) | Foley catheter | 1.23 (0.86, 1.75) | 0.98 (0.7, 1.39) |
| 0.94 (0.6, 1.44) | 0.86 (0.43, 1.68) | 0.81 (0.57, 1.16) | Oral misoprostol | 0.8 (0.57, 1.11) |
| 1.17 (0.79, 1.73) | 1.07 (0.56, 2.08) | 1.02 (0.72, 1.43) | 1.25 (0.9, 1.75) | Vaginal misoprostol |

# Table S16. Head-to-head comparisons of arterial pH

| Dinoprostone | 0.1 (-0.05, 0.25) | 0 (-0.06, 0.05) | 0.09 (-0.06, 0.24) | 0 (-0.04, 0.04) | -0.01 (-0.05, 0.03) |
| --- | --- | --- | --- | --- | --- |
| -0.1 (-0.25, 0.05) | Double-balloon catheter | -0.1 (-0.26, 0.05) | -0.01 (-0.03, 0.02) | -0.1 (-0.25, 0.05) | -0.11 (-0.26, 0.04) |
| 0 (-0.05, 0.06) | 0.1 (-0.05, 0.26) | Double-balloon catheter with oral misoprostol | 0.1 (-0.06, 0.26) | 0 (-0.03, 0.03) | -0.01 (-0.05, 0.03) |
| -0.09 (-0.24, 0.06) | 0.01 (-0.02, 0.03) | -0.1 (-0.26, 0.06) | Foley catheter | -0.1 (-0.25, 0.06) | -0.1 (-0.26, 0.05) |
| 0 (-0.04, 0.04) | 0.1 (-0.05, 0.25) | 0 (-0.03, 0.03) | 0.1 (-0.06, 0.25) | Oral misoprostol | -0.01 (-0.03, 0.01) |
| 0.01 (-0.03, 0.05) | 0.11 (-0.04, 0.26) | 0.01 (-0.03, 0.05) | 0.1 (-0.05, 0.26) | 0.01 (-0.01, 0.03) | Vaginal misoprostol |

# Table S17. Assessment of publication bias for network meta-analysis

| Outcome | Test of Publication Bias Begg’s *P* value | Test of Publication Bias Egger’s *P* value |
| --- | --- | --- |
| Cesarean delivery rate | 0.938 | 0.388 |
| Time from intervention-to-birth (min) | 0.208 | 0.179 |
| Achieving vaginal delivery within 24 hours | 0.602 | 0.525 |
| Bishop score increment | >0.999 | 0.661 |
| Uterine hyperstimulation with fetal heart rate changes | 0.260 | 0.074 |
| Oxytocin augmentation | 0.092 | 0.079 |
| Instrumental delivery | 0.234 | 0.733 |
| Meconium-stained amniotic fluid | 0.828 | 0.908 |
| Chorioamnionitis | 0.371 | 0.377 |
| Postpartum hemorrhage | 0.820 | 0.545 |
| Apgar score <7 in 5 min | 0.758 | 0.806 |
| Apgar score <7 in 1 min | 0.822 | 0.916 |
| Neonatal intensive care unit admission | 0.307 | 0.154 |
| Arterial pH | 0.602 | 0.182 |

# Figure S1. Risk of bias summary.

+: low risk of bias; ?: unclear risk of bias; -: high risk of bias.


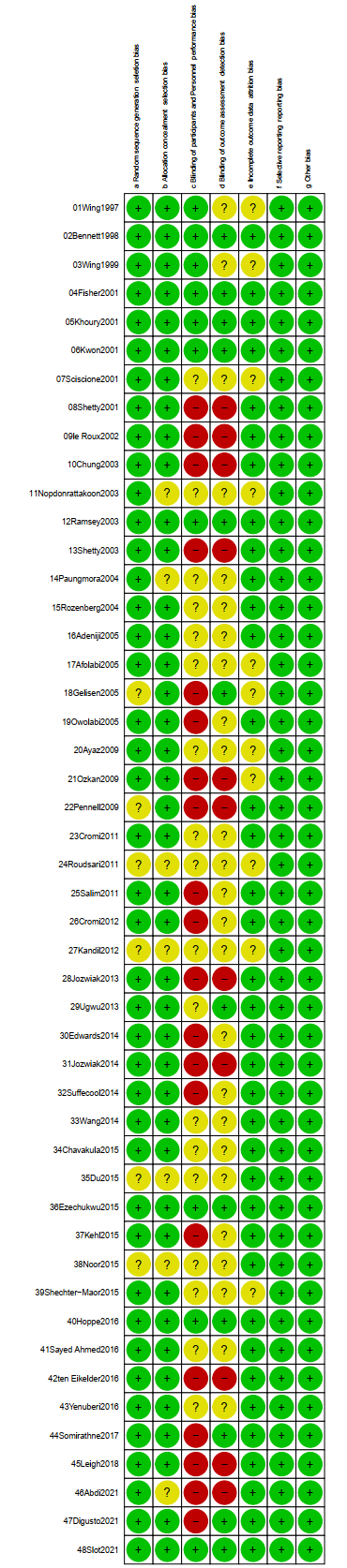


# Figure S2. Risk of bias graph.

+: low risk of bias; ?: unclear risk of bias; -: high risk of bias.


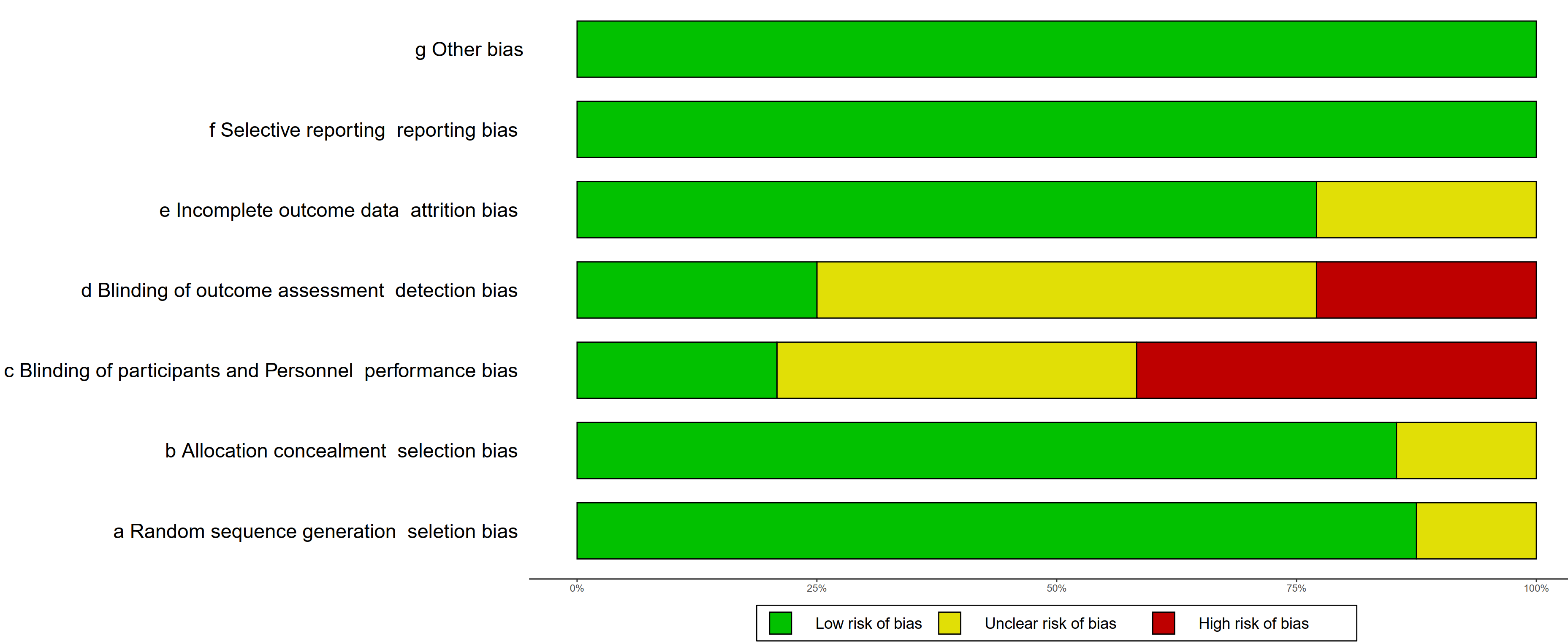


# Figure S3. Network geometry


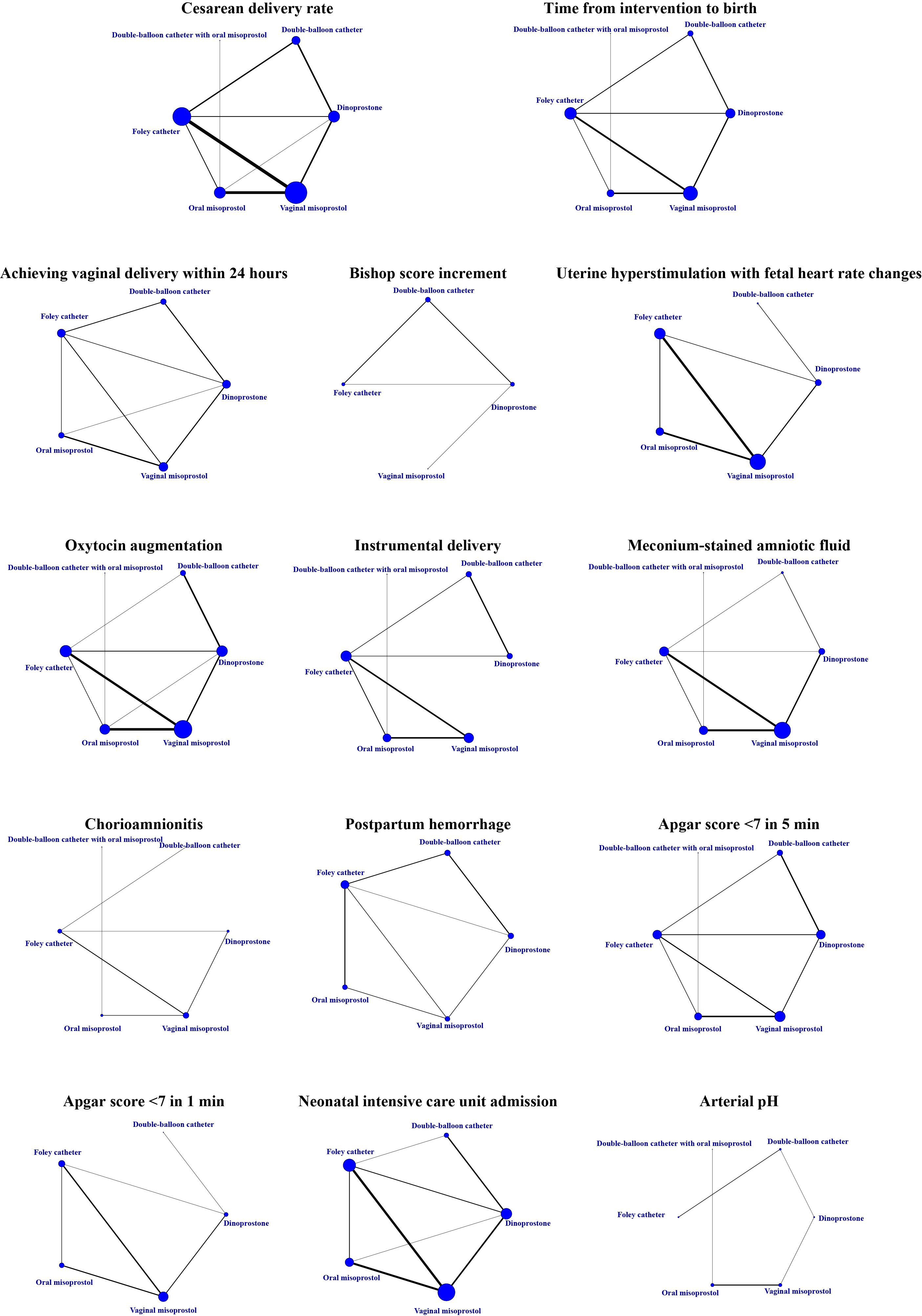


# Figure S4. Inconsistency test of cesarean delivery rate


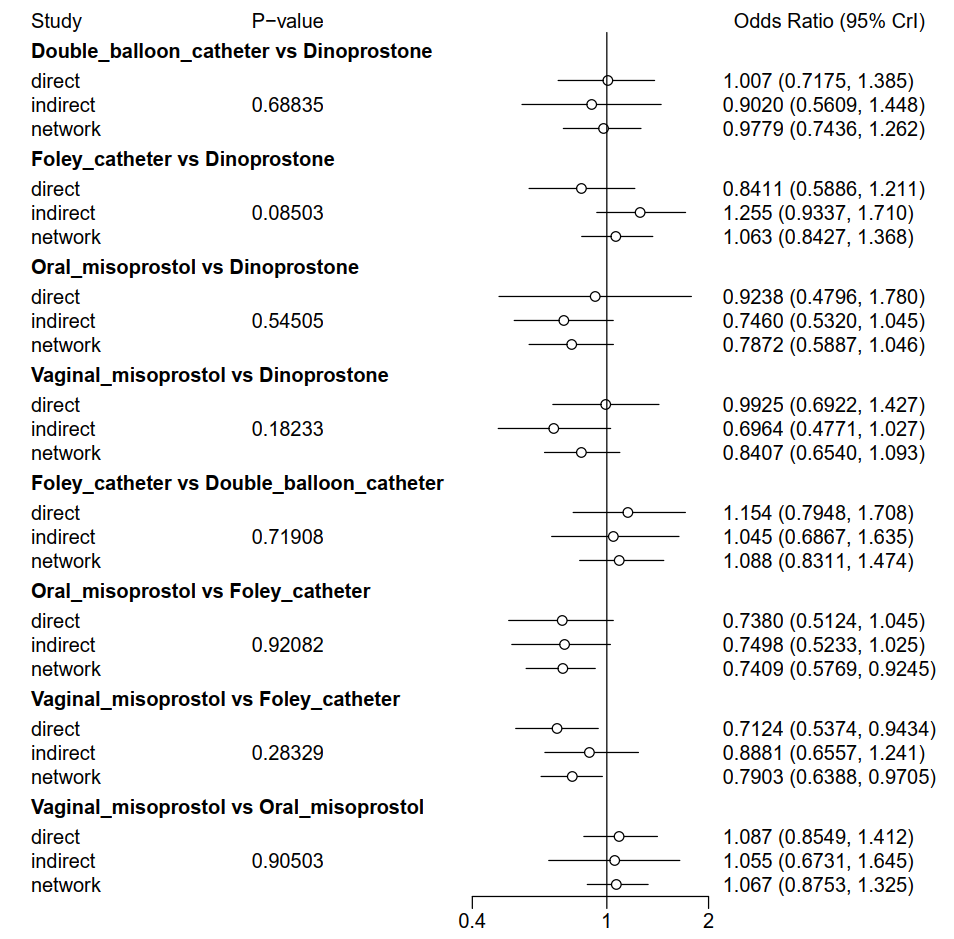


# Figure S5. Inconsistency test of Time from intervention-to-birth


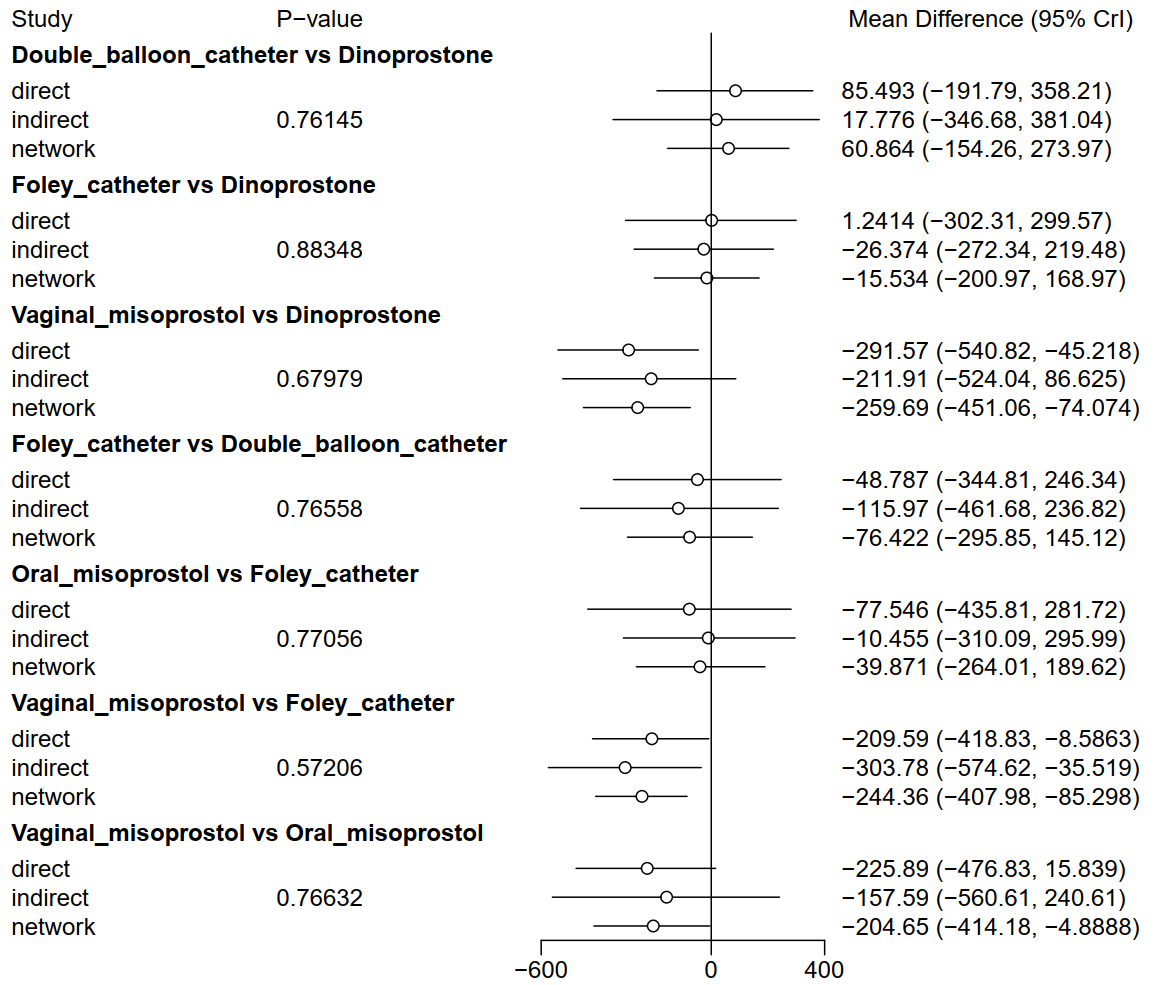


# Figure S6. Inconsistency test of achieving vaginal delivery within 24 hours


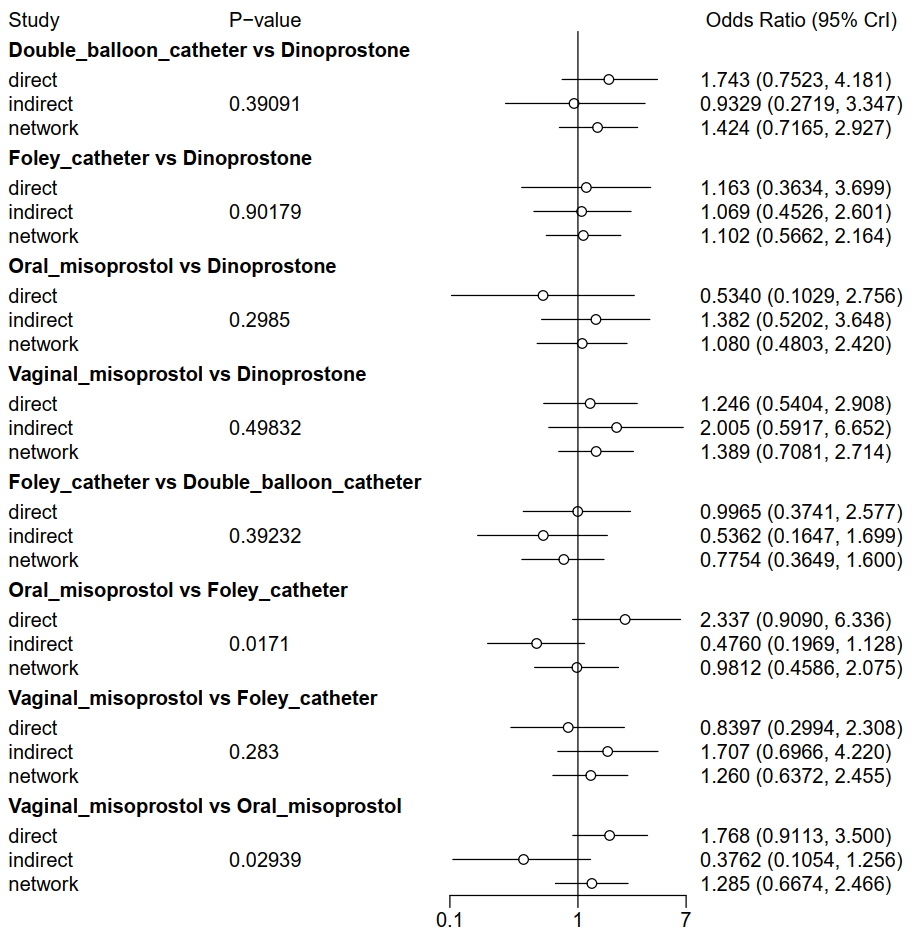


# Figure S7. Inconsistency test of Bishop score increment


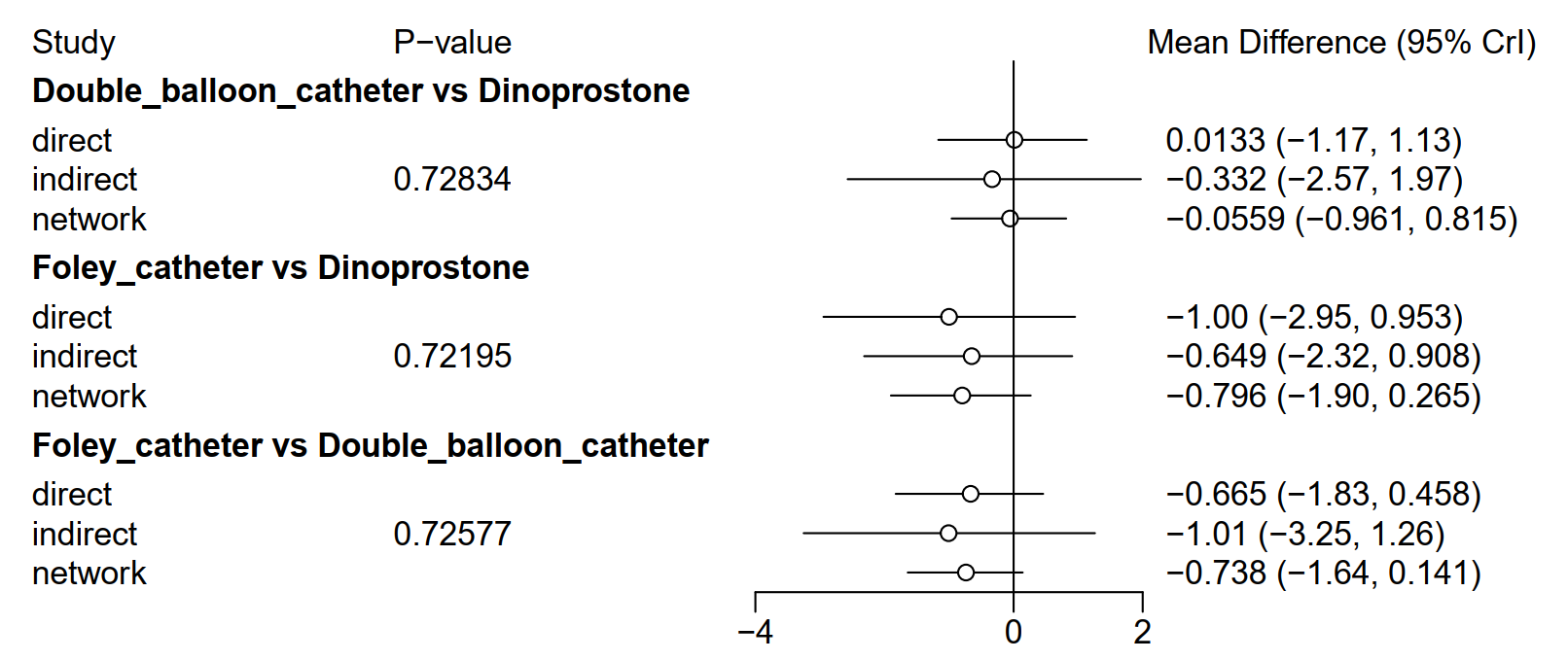


# Figure S8. Inconsistency test of uterine hyperstimulation with fetal heart rate changes


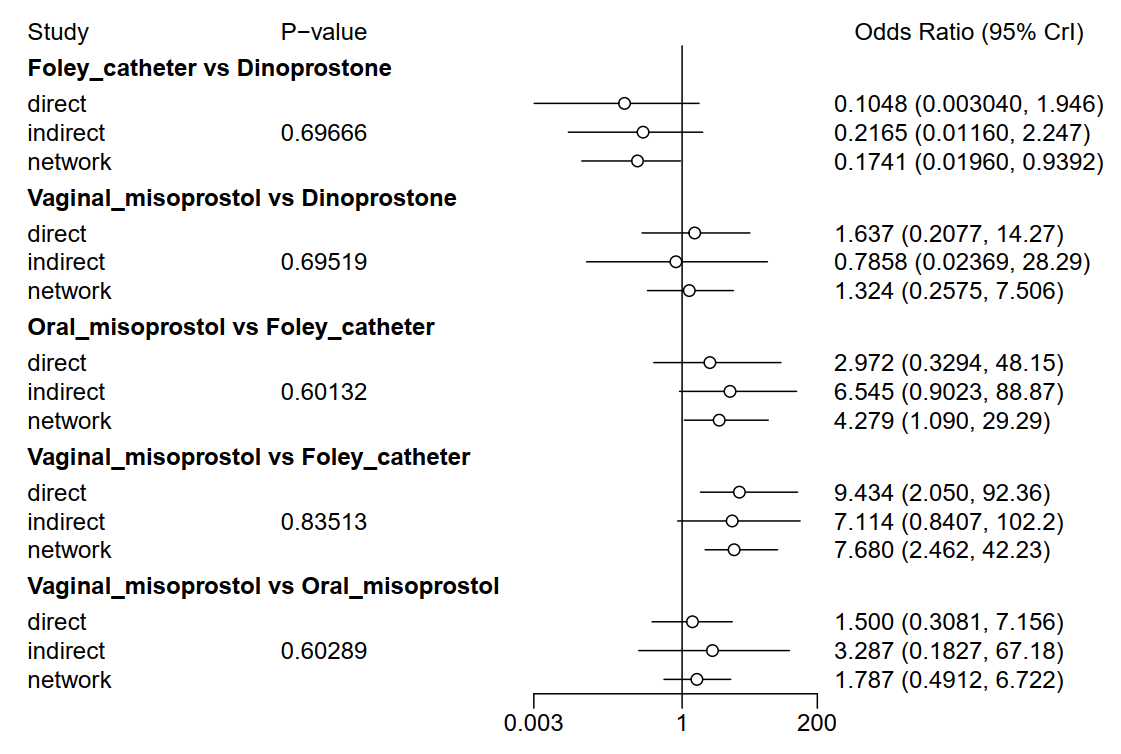


# Figure S9. Inconsistency test of oxytocin augmentation


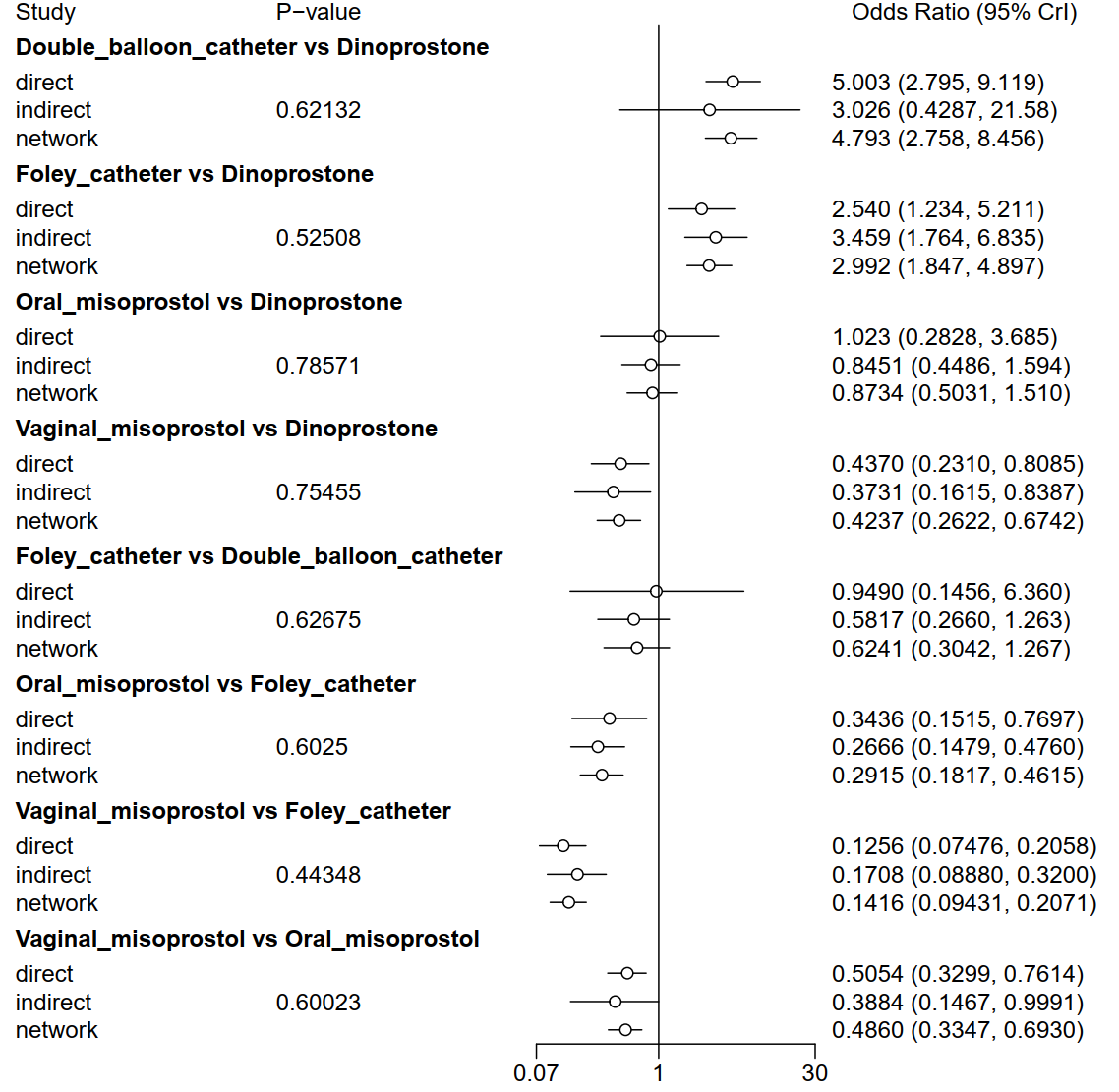


# Figure S10. Inconsistency test of instrumental delivery


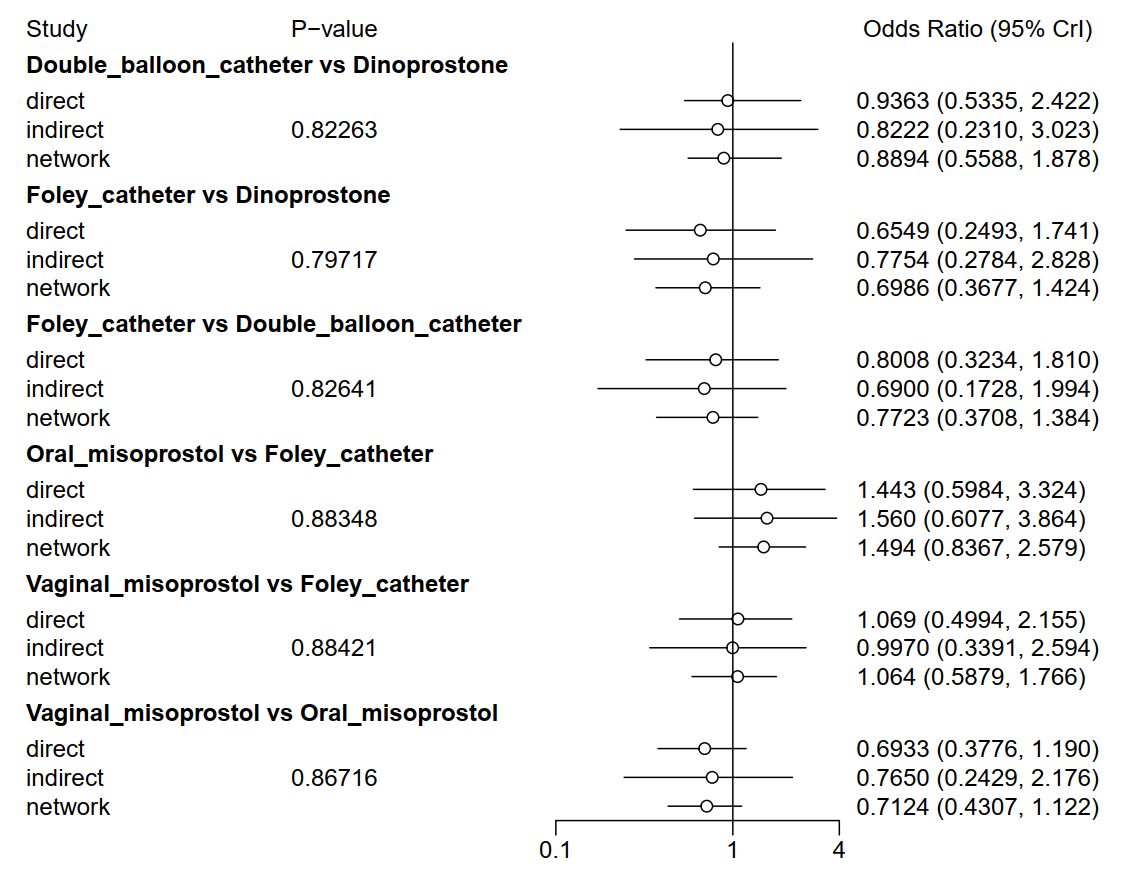


# Figure S11. Inconsistency test of meconium-stained amniotic fluid


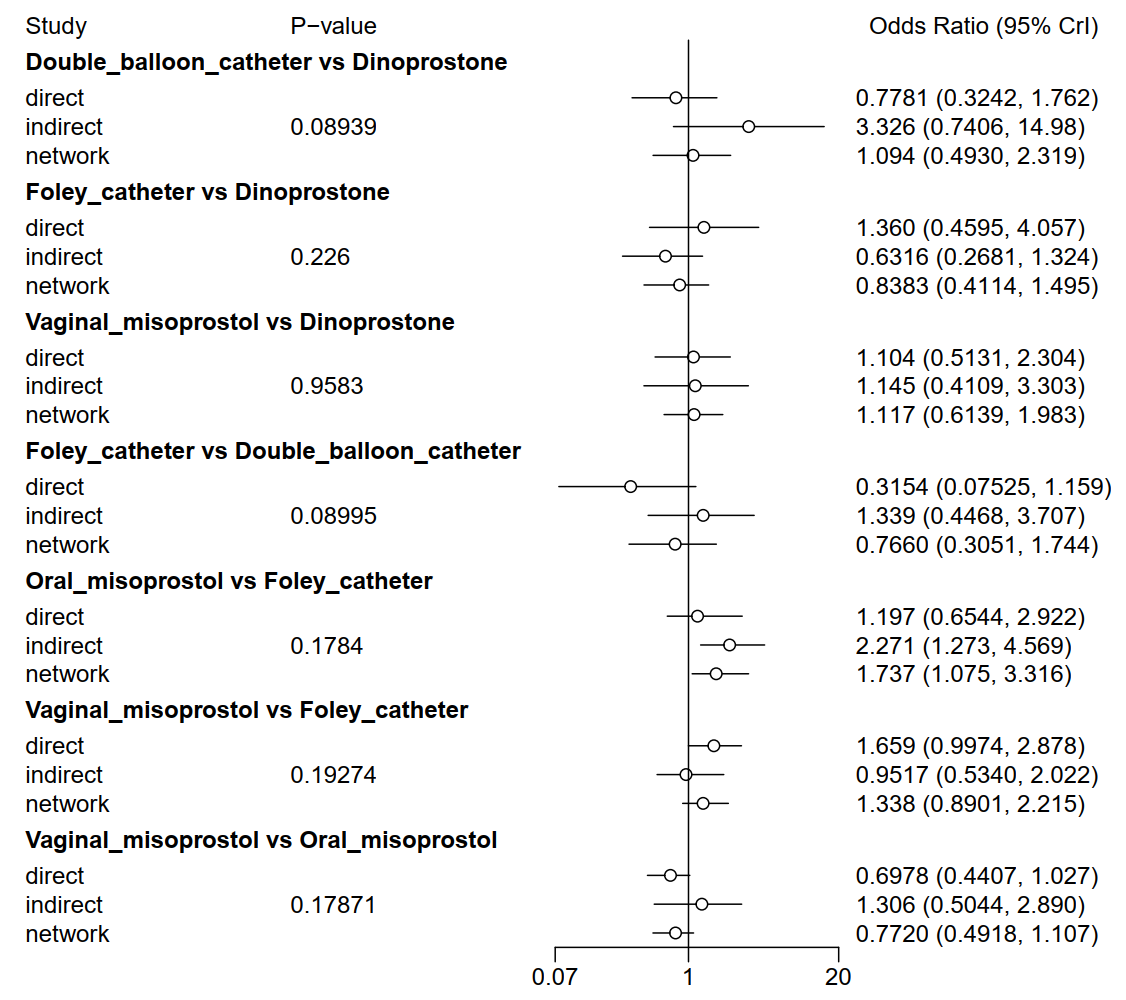


# Figure S12. Inconsistency test of chorioamnionitis


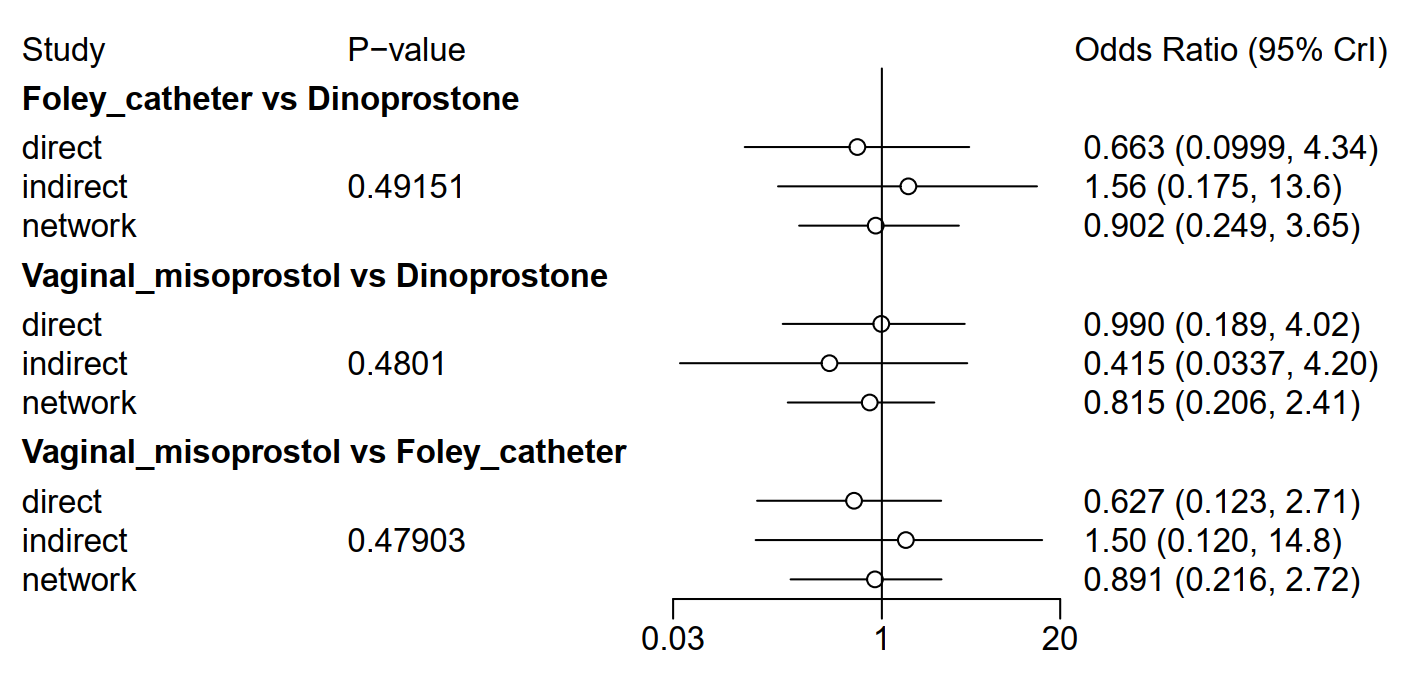


# Figure S13. Inconsistency test of postpartum hemorrhage


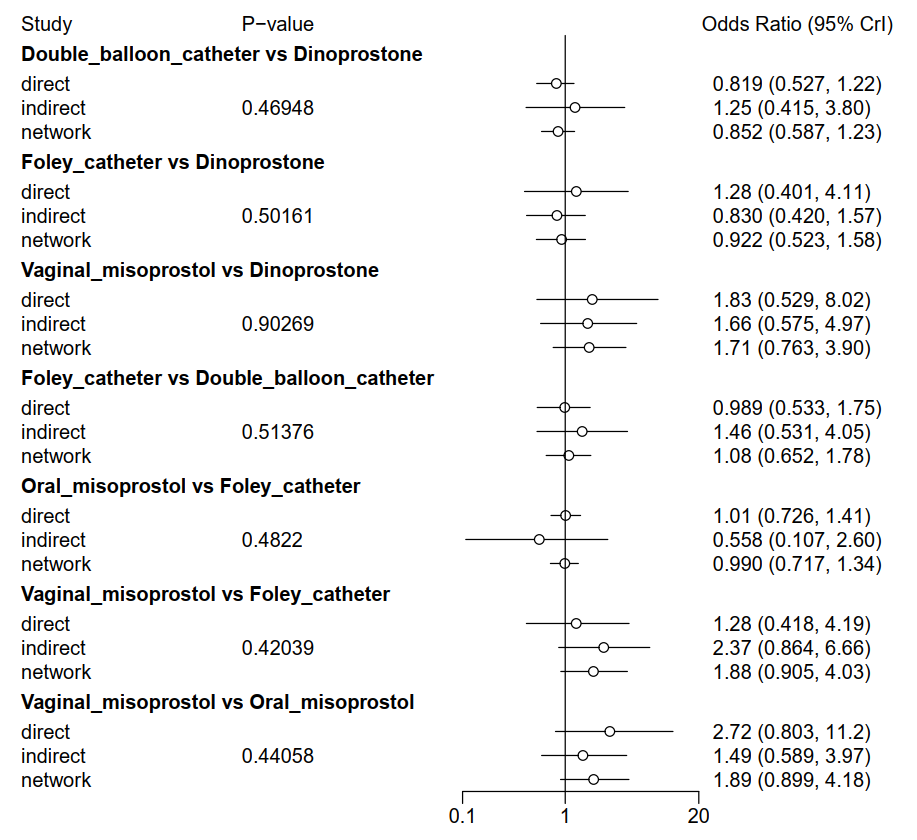


# Figure S14. Inconsistency test of Apgar score <7 in 5 min


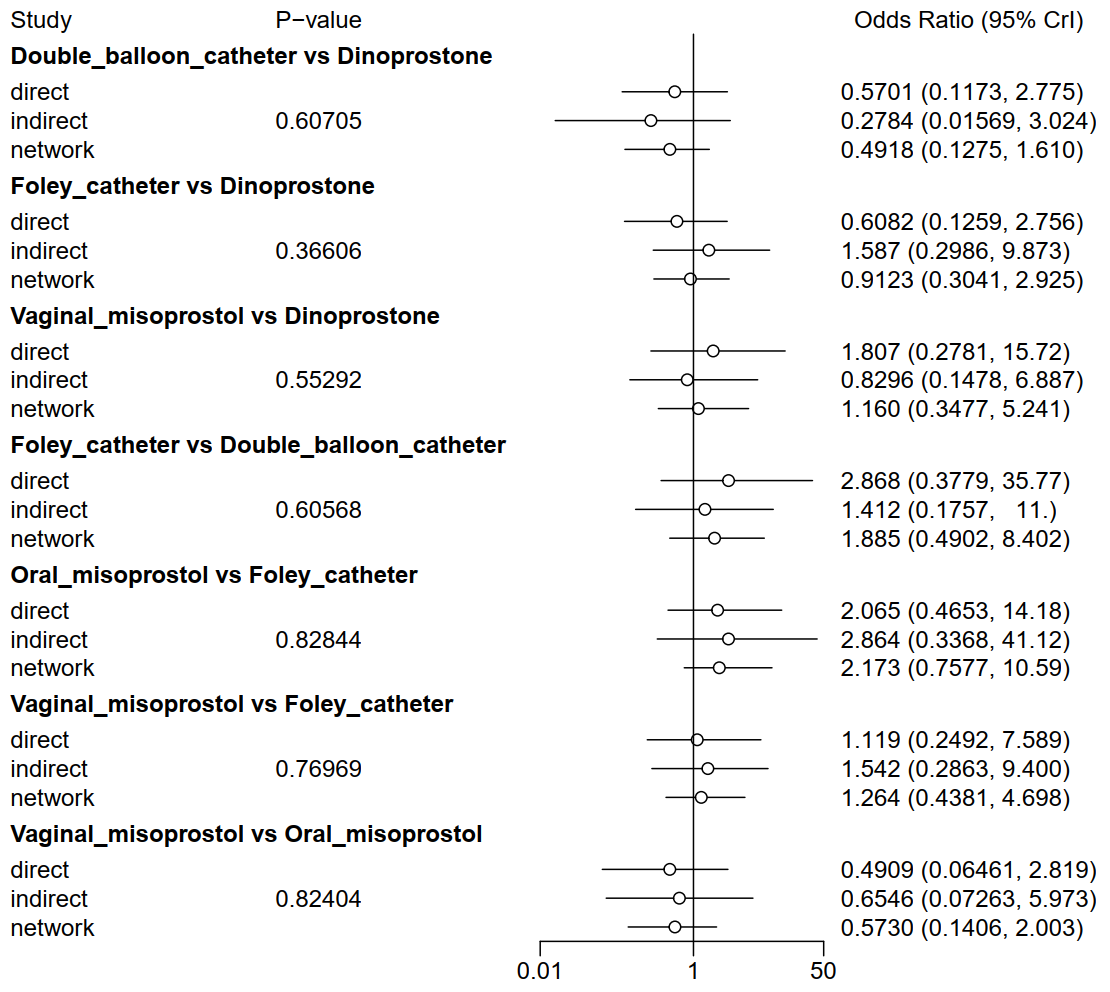


# Figure S15. Inconsistency test of Apgar score <7 in 1 min


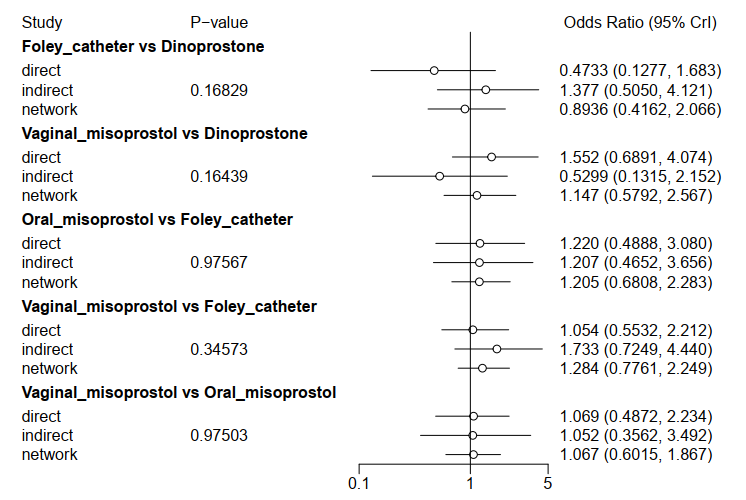


# Figure S16. Inconsistency test of neonatal intensive care unit admission


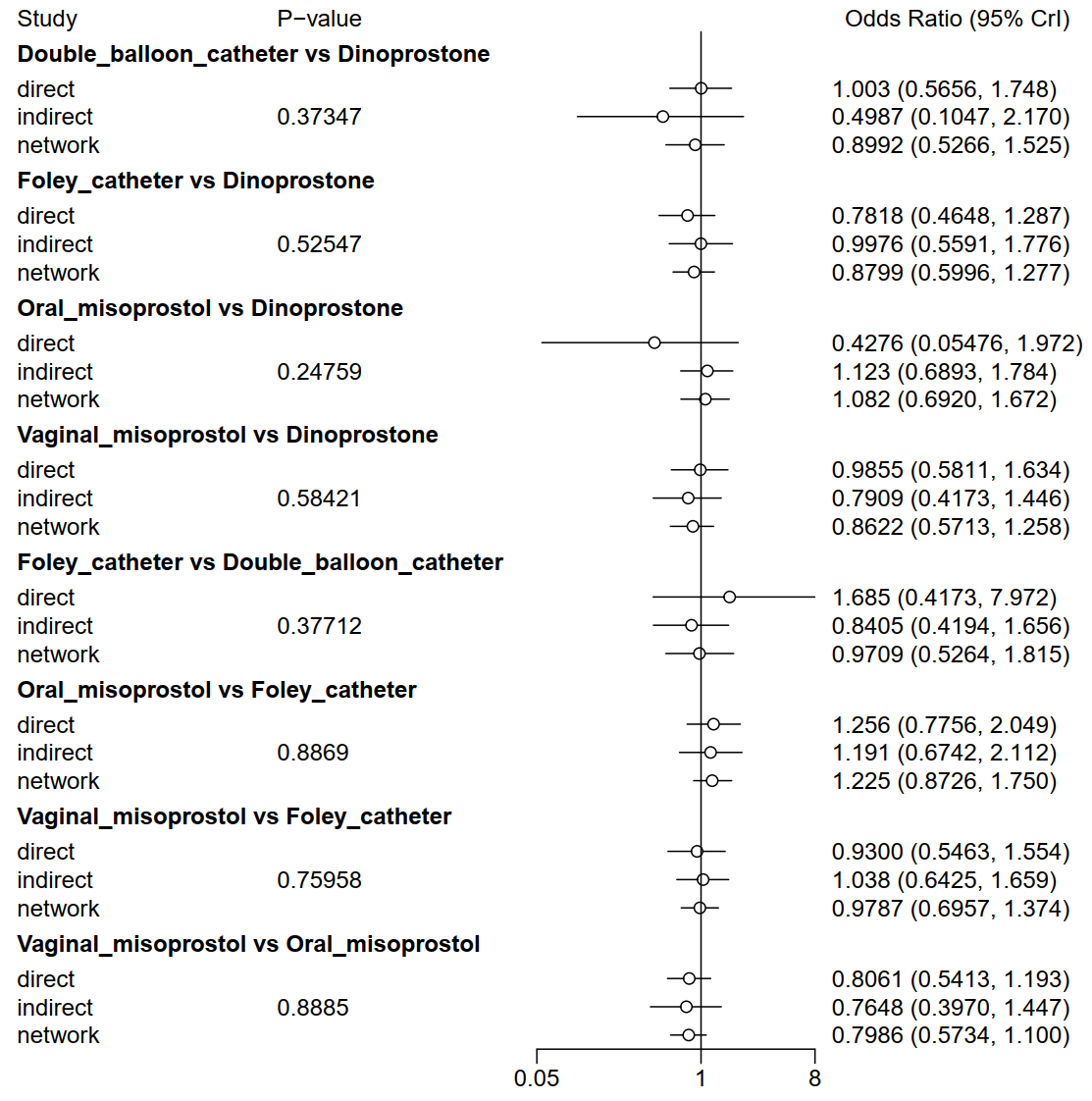


# Figure S17. Funnel plot of primary outcomes


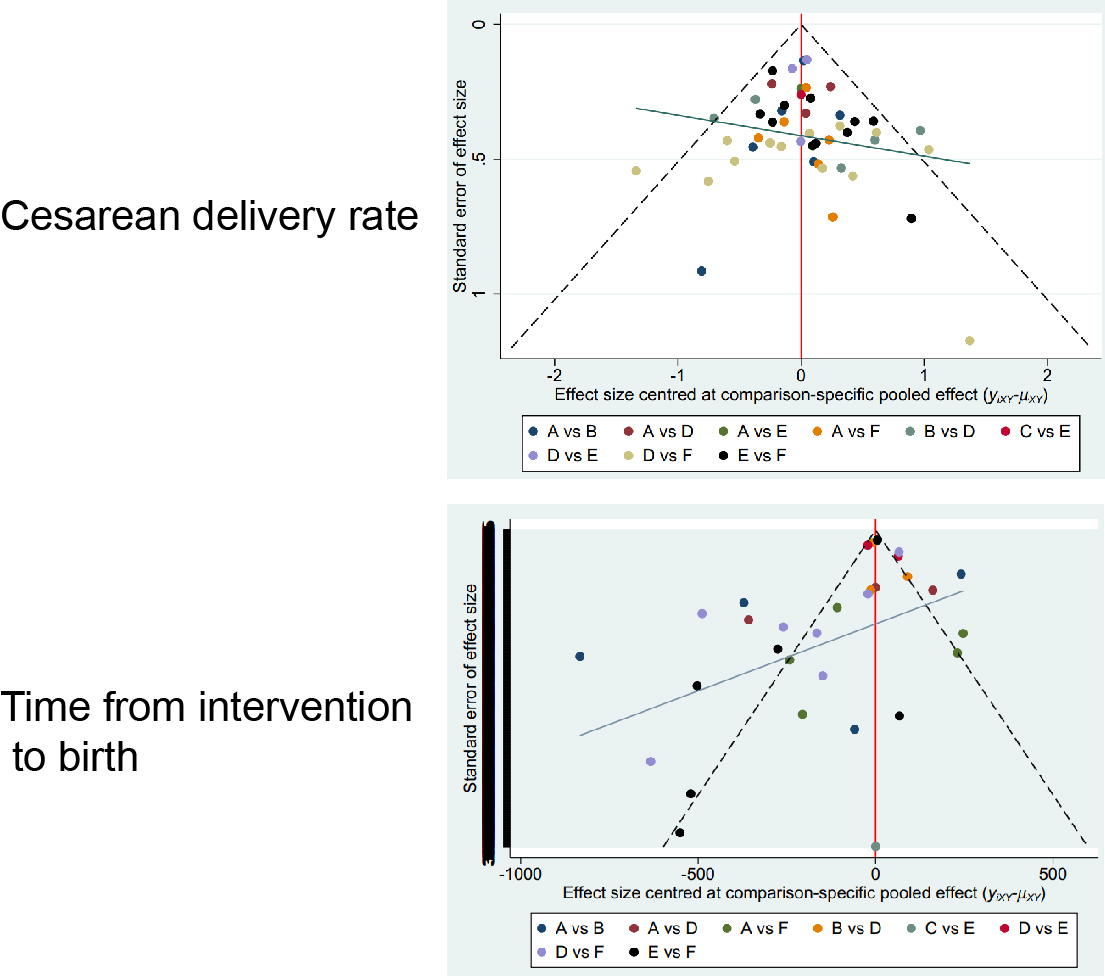


# Figure S18. Funnel plot of secondary outcomes.


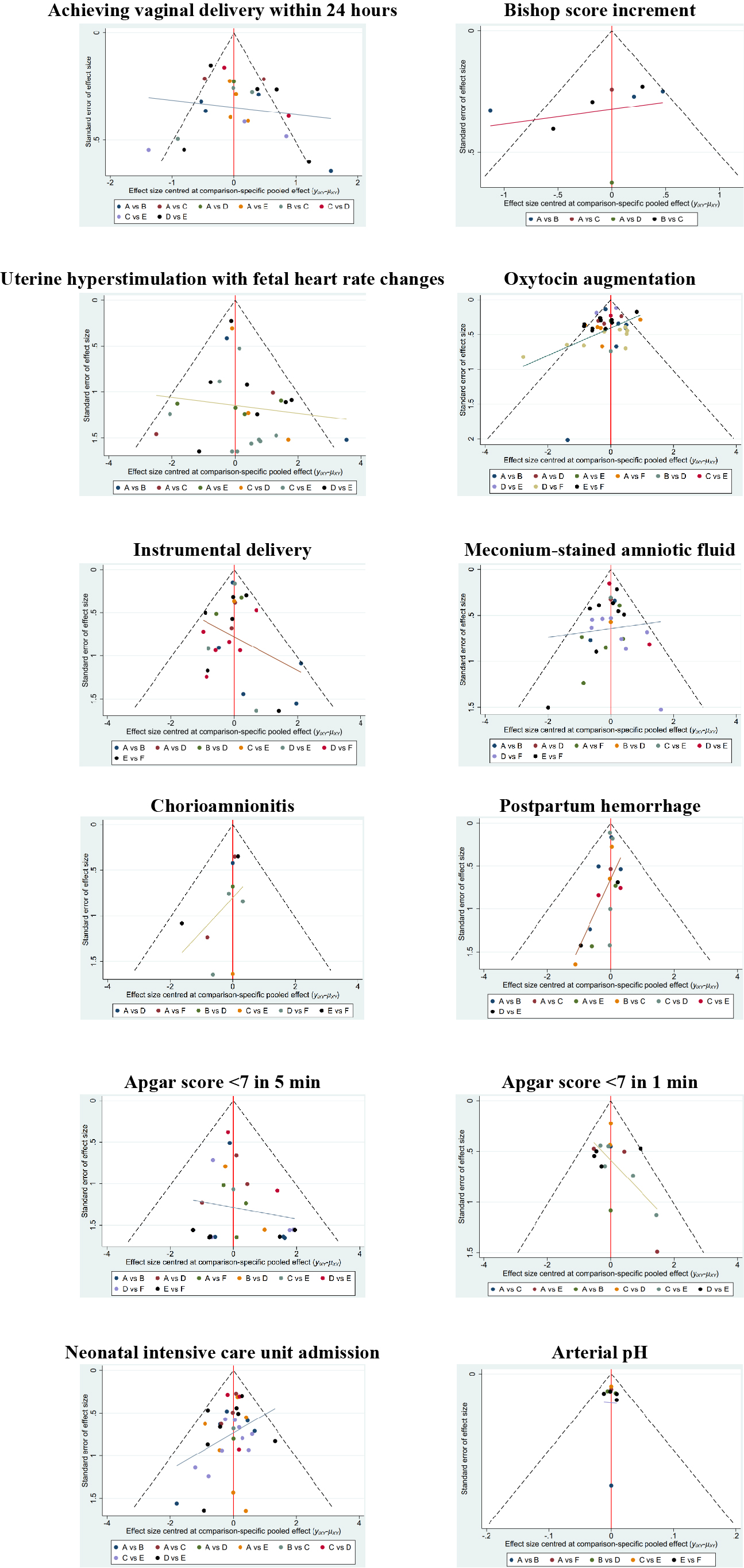

Supplement: Supplementary file 1 — Additional file 1: TableS1. PRISMA Network Meta-analysis Checklist. TableS2. Strategy of this meta-analysis. TableS3. Head-to-head comparisons of cesarean delivery rate. Table S4. Head-to-head comparisons oftime from intervention-to-birth. TableS5. Head-to-head comparisons of achieving vaginal delivery within 24 hours.Table S6. Head-to-head comparisonsof Bishop score increment. Table S7. Head-to-head comparisons of uterine hyperstimulation with fetal heart ratechanges. Table S8. Head-to-headcomparisons of oxytocin augmentation. TableS9. Head-to-head comparisons of instrumental delivery. Table S10. Head-to-head comparisons of meconium-stained amnioticfluid. Table S11. Head-to-headcomparisons of chorioamnionitis. TableS12. Head-to-head comparisons of postpartum hemorrhage. Table S13. Head-to-head comparisons ofApgar score <7 in 5 min. Table S14.Head-to-head comparisons of Apgar score <7 in 1 min. Figure S15. Inconsistency test of Apgar score <7 in 1 min. Table S16. Head-to-head comparisons ofarterial pH. Table S17. Assessmentof publication bias for network meta-analysis. Figure S1. Risk of bias summary. Figure S2. Risk of bias graph. FigureS2. Risk of bias graph. Figure S4. Inconsistency test of cesarean delivery rate. Figure S5. Inconsistency test of Time from intervention-to-birth. Figure S6. Inconsistency test ofachieving vaginal delivery within 24 hours. Figure S7. Inconsistency test of Bishop score increment. Figure S8. Inconsistency test ofuterine hyperstimulation with fetal heart rate changes. Figure S9. Inconsistency test of oxytocin augmentation. Figure S10. Inconsistency test ofinstrumental delivery. Figure S11. Inconsistency test of meconium-stained amniotic fluid. Figure S12. Inconsistency test of chorioamnionitis. Figure S13. Inconsistency test ofpostpartum hemorrhage. Figure S14. Inconsistency test of Apgar score <7 in 5 min. Figure S15. Inconsistency test of Apgar score <7 in 1 min. Figure S16. Inconsistency test ofneonatal intensive care unit admission [file 12884_2022_4988_MOESM1_ESM.docx]
